# Mineralized DNA tetrahedron-structured hydrogels: a dual-functional Scaffold for immunomodulation and bone regeneration

**DOI:** 10.1038/s41413-026-00530-8

**Published:** 2026-05-08

**Authors:** Lan Yao, Jiafei Sun, Zhiqiang Liu, Jiale Liang, Yun Wang, Ye Chen, Ruiqing Wang, Tao He, Yichen Yang, Yao He, Yunfeng Lin, Taoran Tian

**Affiliations:** 1 State Key Laboratory of Oral Diseases & National Center for Stomatology & National Clinical Research Center for Oral Diseases, West China Hospital of Stomatology, Sichuan University, Chengdu, Sichuan China; 2 Stomatology Hospital, School of Stomatology, Zhejiang University School of Medicine, Zhejiang Provincial Clinical Research Center for Oral Diseases, Key Laboratory of Oral Biomedical Research of Zhejiang Province, Cancer Center of Zhejiang University, Engineering Research Center of Oral Biomaterials and Devices of Zhejiang Province, Hangzhou, China; 3 Macao Translational Medicine Center, Macau University of Science and Technology. Taipa, Macau SAR, China; 4 Sichuan Provincial Engineering Research Center of Oral Biomaterials, Chengdu, Sichuan China

**Keywords:** Bone, Endocrine system and metabolic diseases

## Abstract

Clinical limitations of autografts and allografts have driven advances in bone tissue engineering. Emerging biomaterials offer tunable mechanical and bio-regenerative properties for bone reconstruction. DNA hydrogels have attracted increasing attention due to their extracellular matrix–like architecture and excellent cargo-loading capacity. However, their rapid degradation and limited immunomodulatory activity have hindered their long-term efficacy in bone regeneration. To address these limitations, a mineralized tetrahedral framework nucleic acids (tFNAs) hydrogel (Cap-gel) was engineered to integrate early immunoregulation with sustained osteogenic activity. The stable and programmable spatial structure of tFNAs not only promotes macrophage polarization toward the M2 phenotype by presenting immunomodulatory ligands but also serves as a nucleation template for calcium phosphate crystallization, leading to the formation of nano-mineralized structures with controlled morphology. In vitro, Cap-gel promoted osteogenic differentiation via both immune-dependent and independent pathways, while in vivo, it modulated early immune responses and accelerated bone regeneration in a calvarial defect model. In summary, this study introduces a novel tFNA-based mineralized DNA hydrogel system that integrates immunomodulation and osteogenesis, providing a promising strategy for enhanced bone repair in tissue engineering applications.

**Figure:**
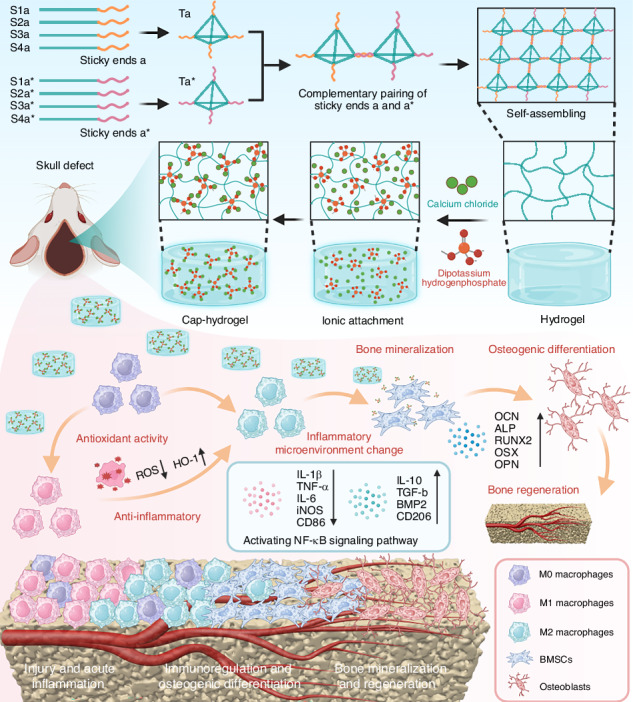
A mineralized DNA hydrogel is constructed by sticky-end self-assembly of tetrahedral framework nucleic acids followed by calcium–phosphate deposition. The hydrogel exhibits antioxidant, immunomodulatory, and osteoinductive properties, enabling sequential regulation of the bone healing microenvironment and effectively promoting cranial defect regeneration

A mineralized DNA hydrogel is constructed by sticky-end self-assembly of tetrahedral framework nucleic acids followed by calcium–phosphate deposition. The hydrogel exhibits antioxidant, immunomodulatory, and osteoinductive properties, enabling sequential regulation of the bone healing microenvironment and effectively promoting cranial defect regeneration

## Introduction

Traumatic injuries, infectious processes, excision of neoplastic lesions, and developmental anomalies frequently result in osseous defects that pose substantial therapeutic challenges. However, the clinical application of conventional repair strategies such as autologous and allogeneic bone grafts is constrained by ethical concerns, species-specific differences, limited donor availability, and the risk of immune rejection.^1–3^ In recent years, bone tissue engineering (BTE) has emerged as a promising alternative, focusing on the design of biomaterials that integrate mechanical strength, biocompatibility, plasticity, and degradability to achieve effective bone regeneration.^4,5^

Hydrogels have attracted significant research attention in BTE owing to their injectability and ability to mimic the extracellular matrix (ECM).^6,7^ These materials provide a three-dimensional environment conducive to cell growth and can also serve as delivery vehicles for drugs, genes, and bioactive molecules,^8,9^ thereby significantly enhancing tissue regeneration efficacy. Currently, hydrogels based on polymers such as hyaluronic acid, gelatin, and collagen (either natural or synthetic) are widely used in bone healing.^10^ However, these hydrogel systems still face significant challenges in immune modulation, degradation rate control, and sustained osteoinductive capacity.^11^ To address these limitations, the development of novel hydrogels capable of combining immunomodulation with osteoinduction has become a central research focus.

Over the past decade, DNA nanomaterials have been increasingly explored in tissue engineering due to their programmability, biodegradability, and molecular recognition capabilities.^12–16^ DNA-based hydrogels have shown promise in drug delivery,^17^ cell delivery,^18,19^ and tissue regeneration.^20^ However, their application in bone defect repair remains limited by three major challenges: (1) the mechanism by which DNA hydrogels regulate the osteoimmune microenvironment is unclear, particularly regarding their role in macrophage polarization; (2) their susceptibility to rapid degradation in vivo hinders long-term regulation of processes such as mineralization; (3) the focus of current strategies on stem cell-loaded hydrogels to promote osteogenesis, which involves high technical complexity and safety risks, thus limiting clinical translation. Therefore, it is imperative to engineer DNA hydrogels with in situ osteoinductive potential, improved mechanical properties, and long-term bone-promoting effects.

Framework nucleic acids (FNAs) represent a prominent class of DNA-based nanostructures with programmable three-dimensional architectures and excellent biocompatibility. Among them, tetrahedral FNAs (tFNAs) have exhibited great potential in regulating diverse cellular processes,^21,22^ including cell behavior,^23^ drug delivery,^24–28^ and biosensing.^29^ Recent studies indicate that tFNAs can be efficiently internalized by macrophages and promote their polarization toward the M2 phenotype, thereby fostering an immune microenvironment favorable for tissue repair.^30–32^ According to osteoimmunology, macrophage polarization influences bone regeneration via cytokine secretion (e.g., TGF-β, BMP2) and angiogenesis regulation. Therefore, integrating tFNAs DNA hydrogels could facilitate spatiotemporal regulation of the immune microenvironment at osseous defect sites while simultaneously supporting osteogenic differentiation.

Although rapid degradation of DNA nanomaterials often compromises their functionality during bone remodeling, particularly in mineralization induction, their inherent phosphodiester backbones exhibit remarkable calcium-binding capacity, which can be leveraged for biomineralization.^33,34^ Notably, pathological calcification processes such as atherosclerosis^35^ and gallstone formation^36^ demonstrate extracellular DNA’s natural involvement in calcium salt deposition. More importantly, non-covalent interactions between DNA and collagen fibers can facilitate localized mineralization in vivo.^37–39^ tFNAs are particularly promising in this regard as their abundant hydrogen-bonding sites and capacity to stabilize supersaturated calcium phosphate solutions make them ideal templates for calcium phosphate crystallization. Building on this, Fan et al. reported a tFNA-mediated strategy that generates nanostructured hydroxyapatite with programmable morphology and enhanced nuclease resistance.^40^ Although the osteoinductive mechanisms of these tFNAs–hydroxyapatite complexes remain to be fully elucidated, these findings lay the theoretical foundation for constructing tFNAs-based mineralized hydrogels with sustained mineralization and osteogenic potential.

In this study, we developed a tFNAs-based mineralized hydrogel system (Cap-gel) to address the limitations of conventional DNA hydrogels in immune modulation and sustained mineralization. By exploiting the structural advantages of tFNAs, this system enables (1) controlled deposition of nanostructured calcium phosphate with programmable morphology within the hydrogel matrix and (2) simultaneous immunomodulation via macrophage polarization. Cap-gel’s dual functionality enables both immediate immune microenvironment regulation and sustained osteoinduction. In vitro experiments demonstrated that Cap-gel directly induces osteogenic differentiation while modulating immune responses to indirectly enhance bone formation. In vivo studies further confirmed effective early-phase immunoregulation (M2 macrophage polarization) and subsequent promotion of mineralization during the remodeling phase. Collectively, this strategy provides (i) a clinically translatable solution to bone defect repair and (ii) a design paradigm for next-generation biomaterials that integrate immunomodulation with controlled biomineralization.

## Results

### Synthesis and characterization of mineralized tFNAs-based hydrogels

**Fig. 1:**
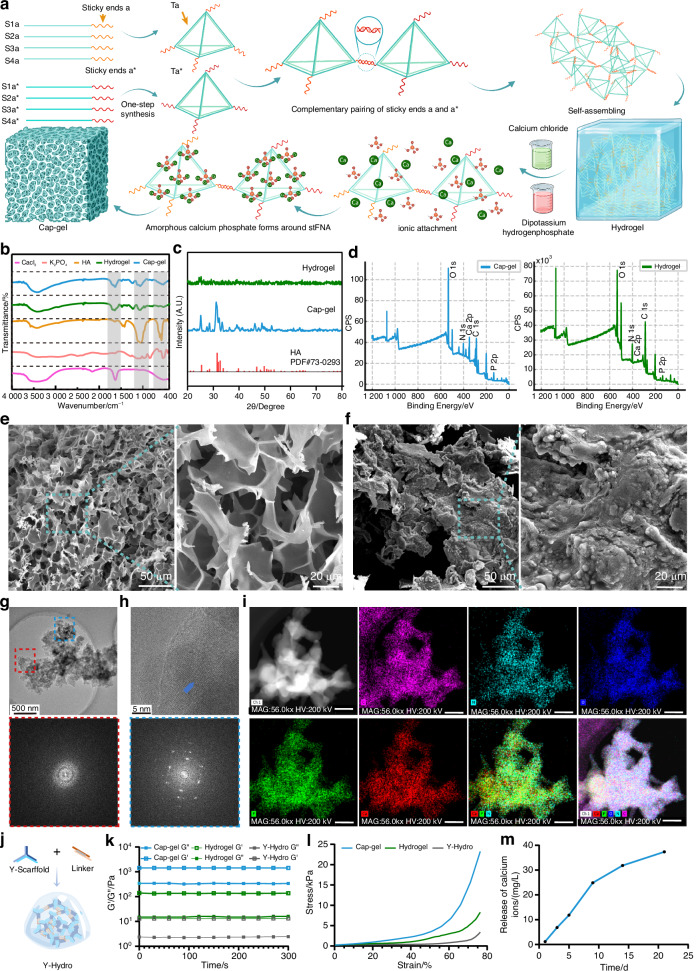
The synthesis and characterization of mineralized tFNAs-based hydrogels. **a** The synthesis schematic of Cap-gel. **b** FTIR spectra of different materials. **c** XRD patterns of Hydrogel and Cap-gel, with HA corresponding PDF reference card number. **d** XPS spectrum of Hydrogel and Cap-gel. **e** SEM images of Hydrogel, the right image is an enlargement of the blue-dotted rectangle in the left image (scale bars: left, 50 µm; right, 20 µm). **f** SEM images of Cap-gel, the right image is an enlargement of the blue-dotted rectangle in the left image (scale bars: left, 50 µm; right, 20 µm). **g** HRTEM images of Cap-gel. Red-dotted rectangle indicating the amorphous state in Cap-gel, as validated by SAED (bar: 500 nm). **h** High-magnification image of the areas in (**g**), showing crystalline nucleation sites (blue arrows) of the crystalline phase, as confirmed by SAED (scale bar: 5 nm). **i** HAADF-STEM and EDS mapping images of Cap-gel are shown, with the elements carbon (C), nitrogen (N), oxygen (O), calcium (Ca), and phosphorus (P) indicated. (scale bar: 400 nm). **j** Schematic diagram of the synthesis of Y-Hydro. **k** Rheological property analysis of Cap-gel, Hydrogel and Y-Hydro. **l** Compressive stress–strain curve of Cap-gel, Hydrogel and Y-Hydro. **m** The release concentration of calcium of Cap-gel in 21 days

Leveraging the programmable nature of tFNAs, we designed two complementary building blocks (Ta and Ta*; sequences in Table S1) with specific sticky ends. These units were synthesized in a single step and combined at a 1:1 molar ratio to construct the tFNA-based hydrogel system (termed Hydrogel). Mineralization was subsequently achieved through alternating incubation with calcium chloride (CaCl_2_) and dipotassium hydrogen phosphate (K_2_HPO_4_) solutions, yielding the mineralized tFNA-based hydrogel system (Cap-gel) (Fig. 1a).

Fourier-transform infrared spectroscopy (FTIR) analysis (Fig. 1b) revealed that the hydroxyapatite (HA) standard (orange curve) exhibited characteristic calcium phosphate mineralization vibrational modes, with distinct absorption bands at 1 100 cm^−1^ (ν_3_PO_4_^3-^ asymmetric stretching) and 500 cm^−1^ (ν_3_PO_4_^3-^ bending), confirming mineral composition. AdditionallyWhen comparing the spectra of them, Cap-gel demonstrated enhanced absorption peaks in the mineralization-related regions (500 cm^−1^ and 1 100 cm^−1^), while maintaining the organic matrix signature (C-H bending vibration features around 1 600 cm^−1^), similar to Hydrogel, indicating successful calcium phosphate deposition on the Hydrogel template. To further characterize the crystalline structure differences before and after mineralization, X-ray diffraction (XRD) analysis was performed. (Fig. 1c). In the XRD spectrum, Cap-gel (blue curve) exhibited well-defined diffraction peaks, especially in the 2θ range of approximately 20° to 40°,that closely matched the reference pattern for HA. The XRD spectrum of the unmineralized Hydrogel was relatively smooth, lacking characteristic peaks, and exhibited lower crystallinity. In contrast, Cap-gel exhibited a stronger crystalline structure and mineralization characteristics, proving that Cap-gel forms stable calcium phosphate compounds during the mineralization process. The X-ray photoelectron spectroscopy (XPS) analysis revealed stronger Ca 2p and P 2p signals in Cap-gel (blue curve) compared to Hydrogel, confirming successful calcium phosphate formation. In contrast, the similar intensities of N 1 s and C 1 s peaks in both groups suggest that the organic components remained stable and the mineralization process did not alter the original hydrogel template (Fig. 1d and Fig. S1). Scanning electron microscopy (SEM) characterization of lyophilized samples provided direct morphological evidence: while the Hydrogel exhibited a loosely organized, uniform porous structure with smooth pore walls and no observable crystalline features (Fig. 1e), consistent with its polymeric composition, Cap-gel displayed a markedly rougher surface topography characterized by irregular particle deposition and distinct crystalline formations (Fig. 1f), directly evidencing successful mineralization.

High-resolution transmission electron microscopy (HRTEM) analysis of Cap-gel revealed a heterogeneous microstructure comprising both amorphous hydrogel materices and well-defined crystalline nucleation sites (Figs. 1g, h). The amorphous hydrogel matrix, marked by red-dotted rectangles, displayed no discernible lattice fringes, with Selected area electron diffraction (SAED) patterns confirming its non-crystalline nature through the absence of diffraction rings. In contrast, localized crystalline domains (indicated by blue arrows) exhibited distinct nucleation features, indexable to characteristic polycrystalline diffraction rings in SAED. Moreover, HRTEM analysis revealed well-ordered lattice fringes with consistent interplanar spacing, demonstrating high crystallinity of the mineralized phase. Quantitative analysis revealed an average interplanar spacing of 0.330 7 ± 0.014 2 nm (Fig. S2), which is consistent with the characteristic d-spacing observed in tFNA-mineralized crystals.^40^ Elemental mapping via energy-dispersive X-ray spectroscopy (EDS) revealed that calcium-phosphate deposition preferentially occurred in oxygen-enriched regions while maintaining uniform carbon and nitrogen distribution, suggesting oxygen-mediated mineralization without compromising the organic hydrogel matrix (Fig. 1i). These results collectively demonstrate the successful formation of crystalline Ca-P phases within the amorphous hydrogel network through controlled mineralization.

Further, rheological and mechanical tests were conducted to characterize the mechanical strength of Cap-gel. Compared to the common Y-type DNA hydrogel form (Y-Hydro) (Fig. 1j), both Hydrogel and Cap-gel showed higher storage moduli (G’), with Cap-gel outperforming Hydrogel (Fig. 1k), highlighting its superior structural stability and mechanical strength. Consistently, compressive stress–strain testing revealed that Cap-gel achieved a compressive strength of 23.25 ± 2.57 kPa, substantially higher than Hydrogel (7.68 ± 1.25 kPa) and Y-Hydro (2.05 ± 0.89 kPa), confirming mechanical reinforcement after mineralization. (Fig. 1l). In vitro ion release experiments confirmed that calcium ions (Ca^2+^) from Cap-gel were continuously released for at least 21 days, exhibiting a trend of slow early release, increasing release rates in the middle phase, and leveling off in the later phase. This release pattern aligns with the calcium demand in the bone regeneration microenvironment and provides strong evidence for Cap-gel as a mineral reservoir (Fig. 1m). Additionally, the degradation of DNA in vitro was monitored using a UV spectrophotometer (Fig. S3). DNA degradation in the Hydrogel reached a plateau after 120 h, while the degradation rate in the Cap-gel was slightly slower. This is presumably due to the protective effect of the mineralized layer, which may shield the DNA from enzymatic digestion.

### In vitro alleviation of inflammation by mineralized and non-mineralized tFNAs-based hydrogels

**Fig. 2:**
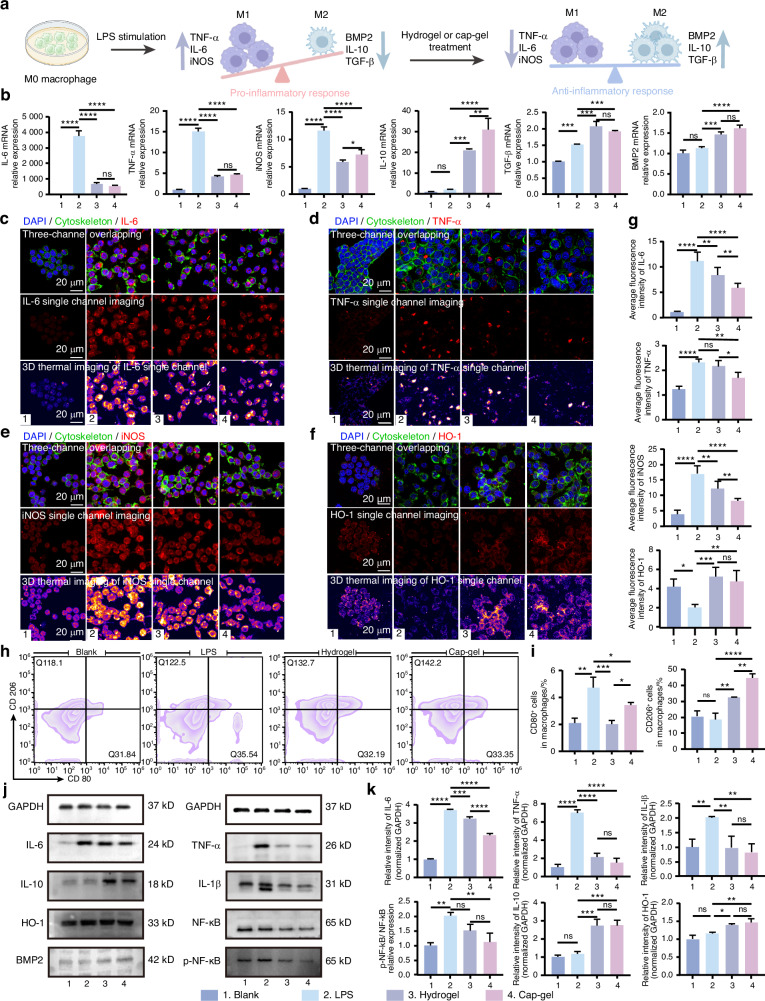
Mineralized and non-mineralized tFNAs-based hydrogels alleviate inflammation in vitro. **a** Schematic diagram of Hydrogel or Cap-gel regulating macrophage polarization. **b** qPCR results of different treatment groups. Immunofluorescence detection images of IL-6 (**c**), TNF-α (**d**), iNOS (**e**), HO-1 (**f**) in macrophages (scale bar: 20 μm). **g** Statistical results of relative fluorescence intensity of different proteins in (**c**–**f**). **h** Flow cytometry results of macrophage polarization status. **i** Statistical results of the proportion of CD80^+^ or CD206^+^ cells in macrophages. **j** Western blot results of various proteins in macrophages. **k** Statistical analysis of the relative expression levels of various proteins normalized to GAPDH. Data are presented as mean ± SD (*n* ≥ 3), *P*-values are calculated using one-way ANOVA, *P < 0.05, **P < 0.01, ***P < 0.001, ****P < 0.000 1, ns no significance

To systematically investigate the immunomodulatory properties of Cap-gel and Hydrogel, we established a well-characterized in vitro inflammation model using LPS-stimulated macrophages (Fig. 2a). Quantitative PCR analysis of inflammatory markers across treatment groups (Fig. 2b) revealed that both Cap-gel and Hydrogel significantly downregulated the expression of pro-inflammatory cytokines (IL-6, TNF-α, and iNOS) while upregulating anti-inflammatory factors (IL-10, TGF-β, and BMP2) compared to LPS-stimulated controls, consistent with macrophage polarization toward the M2 phenotype. Notably, Cap‑gel upregulated BMP2, indicating that they not only modulate the inflammatory environment but also potentially promote osteogenesis indirectly via enhanced BMP2 secretion.

Furthermore, immunofluorescence staining results corroborated the qPCR findings (Figs. 2c–g). LPS-induced over-expression of IL-6, TNFα, and iNOS was markedly suppressed by both Cap-gel and Hydrogel treatments. In addition to anti-inflammatory effects, both Cap-gel and Hydrogel alleviated cellular oxidative stress, as evidenced by decreased intracellular reactive oxygen species (ROS) accumulation (Fig. S4) and upregulated expression of the antioxidant factor HO-1 (Fig. 2f).

Both Cap-gel and Hydrogel treatments modulated macrophage polarization (Fig. 2h). Flow cytometry analysis showed that both treatments reduced M1 macrophages (CD80^+^) and increased M2 macrophages (CD206^+^) (Fig. 2i). Western blot (WB) analysis of macrophage lysates performed after treatments further elucidated the anti-inflammatory and antioxidant mechanisms (Fig. 2j). Quantitative analysis (Fig. 2k) demonstrated significant downregulation of pro-inflammatory cytokines (IL-6, TNF-α, and IL-1β) in both groups compared to controls, with Cap-gel showing superior suppression of IL-6. Concurrently, elevated expression of anti-inflammatory IL-10 and antioxidant HO-1 was observed.

Furthermore, consistent with the PCR results, Cap-gel effectively induced macrophages to secrete BMP2 (Fig. 2i and Fig. S5), enabling a critical functional shift within the osteoimmune microenvironment. Specifically, macrophage-derived BMP2 acts as a paracrine osteogenic signal that directly targets neighboring mesenchymal stem cells, thereby initiating the osteogenic differentiation cascade. This pathway effectively bridges the early immunomodulatory phase with the subsequent bone-forming phase, ensuring a seamless transition from inflammation resolution to the activation of regenerative signaling. This indicates that Cap-gel not only suppresses inflammation but also actively directs macrophage polarization toward a reparative phenotype, empowering these immune cells to orchestrate downstream osteogenesis.

Previous studies have confirmed that tFNAs primarily regulate inflammatory responses via the NF-κB signaling pathway.^41^ In line with this, our results demonstrate that Cap-gel significantly suppresses NF-κB activation, as evidenced by reduced phosphorylation and a decreased p-NF-κB/NF-κB ratio, thereby attenuating the inflammatory cascade and contributing to its immunomodulatory function.

### Direct regulation of osteogenic activity in BMSCs by mineralized tFNAs-based hydrogels in vitro

**Fig. 3:**
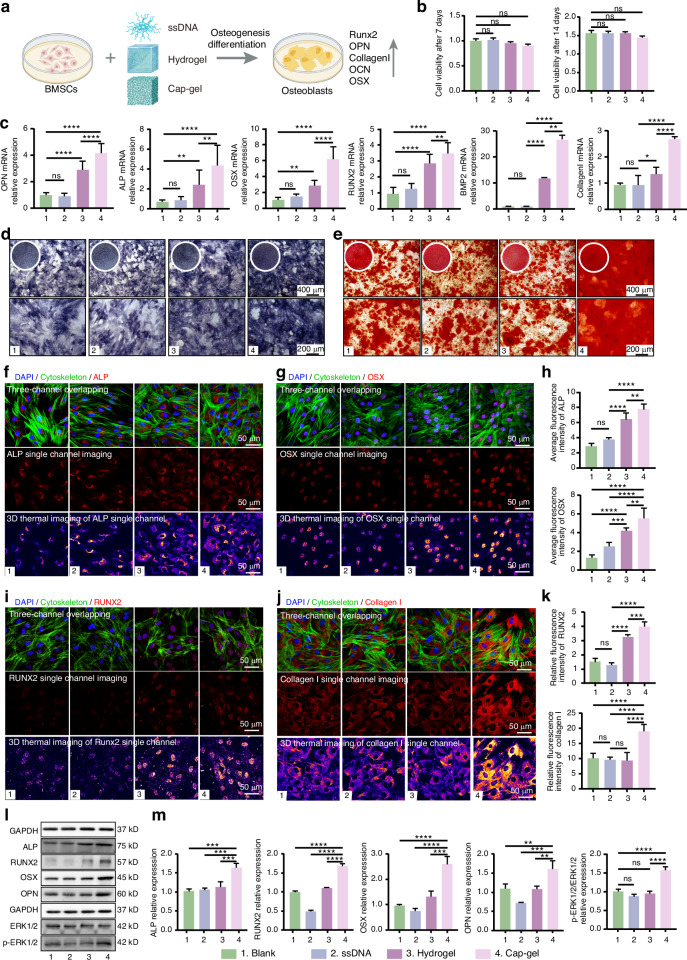
Mineralized tFNAs-based hydrogels directly regulate the osteogenic activity of BMSCs in vitro. **a** Schematic diagram of treating BMSCs. **b** Cell viability of BMSCs co-cultured with Cap-gel for 7 and 14 days. **c** qPCR results of different treatment groups. **d** ALP staining results after 7 days of culture in different treatment groups. **e** Alizarin red staining results after 14 days, scale bars: 400 μm (upper row), 200 μm (lower row). The complete image of the corresponding orifice plate is placed in the upper left corner. **f** Immunofluorescence detection images of ALP in BMSCs (scale bar: 50 μm). **g** Immunofluorescence detection images of OSX in BMSCs (scale bar: 50 μm). **h** Statistical results of relative fluorescence intensity of different proteins in (**f**, **g**). **i** Immunofluorescence detection images of RUNX2 in BMSCs (scale bar: 50 μm). **j** Immunofluorescence detection images of Collagen I in BMSCs (scale bar: 50 μm). **k** Statistical results of relative fluorescence intensity of different proteins in (**i**, **j**). **l** Western blot results of various proteins in BMSCs. **m** Statistical analysis of the relative expression levels of various proteins normalized to GAPDH. Data are presented as mean ± SD (*n* = 3), *P*-values are calculated using one-way ANOVA, *P < 0.05, **P < 0.01, ***P < 0.001, ****P < 0.000 1, ns no significance

After confirming the macrophage polarization-modulating properties of Cap-gel and Hydrogel toward the anti-inflammatory phenotype, we next investigated their direct effects on bone marrow stem cell (BMSC) osteogenic differentiation in vitro (Fig. 3a). Considering the longer culture period, we first assessed the cytotoxicity of Cap-gel. CCK-8 assays showed that after 7 and 14 days of co-culture, Cap-gel showed no significant inhibition of cell viability (Fig. 3b), indicating favorable biocompatibility for long-term applications. Following 7 days of osteogenic induction, Cap-gel significantly enhanced the expression of key osteogenic markers: alkaline phosphatase (ALP), osteopontin (OPN), runt-related transcription factor 2 (RUNX2), osterix (OSX), bone morphogenetic protein 2 (BMP2), and collagen type I (Collagen I). The elevated levels were superior to those in the single-stranded DNA (ssDNA) and Hydrogel groups. Notably, BMP2, an essential mediator of osteoblast differentiation^42,43^ and a key regulator in bone formation, was upregulated nearly 30-fold in the Cap-gel group, underscoring its strong osteogenic and regenerative potential.

ALP staining of BMSCs cultured for 7 days revealed the most intense and uniformly distributed staining in the Cap-gel group, suggesting enhanced early osteogenic differentiation of BMSCs (Fig. 3d and Fig. S5). 14 days of osteogenic stimulation resulted in markedly increased mineralized nodule formation in Cap-gel samples, as evidenced by alizarin red staining (Fig. 3e) and semi-quantitative mineralization levels of calcium nodules (Fig. S6). In comparison, the three comparator groups displayed only scant mineralized nodules, with comparable mineralization levels across them. These results indicate that Cap-gel possesses a clear advantage in driving both osteogenic differentiation and mineralization. Immunofluorescence imaging (Fig. 3f–k) confirmed the osteogenic potential of Cap-gel at the protein level, consistent with the qPCR results. Both methods demonstrated consistent upregulation of ALP, OSX, RUNX2, OPN (Fig. S7), and Collagen I.

Finally, osteogenic protein expression and ERK pathway phosphorylation in BMSCs were analyzed by Western blot (Fig. 3l). Protein levels of ALP, OPN, RUNX2, and OSX were significantly increased following Cap‑gel treatment, differing markedly from the other groups (Fig. 3m). Elevated phosphorylated ERK1/2 was detected only in the Cap‑gel group. As a key effector of the MAPK pathway, ERK1/2 can be activated independently by Ca^2+^ via the calcium‑sensing receptor (CaSR) on BMSCs.^44–46^ Based on these results and the sustained Ca^2+^ release from Cap‑gel, we propose that Cap‑gel directly promotes osteogenic differentiation and mineralization in BMSCs through Ca^2+^‑mediated activation of the ERK/MAPK pathway.

### Indirect regulation of osteogenic activity in BMSCs by mineralized tFNAs-based hydrogels in vitro

Based on the above results, Cap-gel can both suppress inflammatory expression in macrophages and promote osteogenic differentiation of BMSCs. However, whether it can further exert synergistic effects on bone regeneration in the context of macrophage–BMSC crosstalk remained unclear. Current osteoimmunology research indicates that pro-inflammatory mediators (e.g., IL-1β, IL-6, TNF-α) within an inflammatory milieu exacerbate bone resorption and suppress osteogenesis, primarily via the activation of NF-κB cascades.^47,48^ In contrast, M2-polarized macrophages secrete anti-inflammatory factors to inhibit osteoclast activity while simultaneously releasing osteogenic factors such as BMP2 to promote osteoblast activation.^49^ Therefore, immune-modulating biomaterials are increasingly recognized for their enhanced osteoinductive potential.^50^

**Fig. 4:**
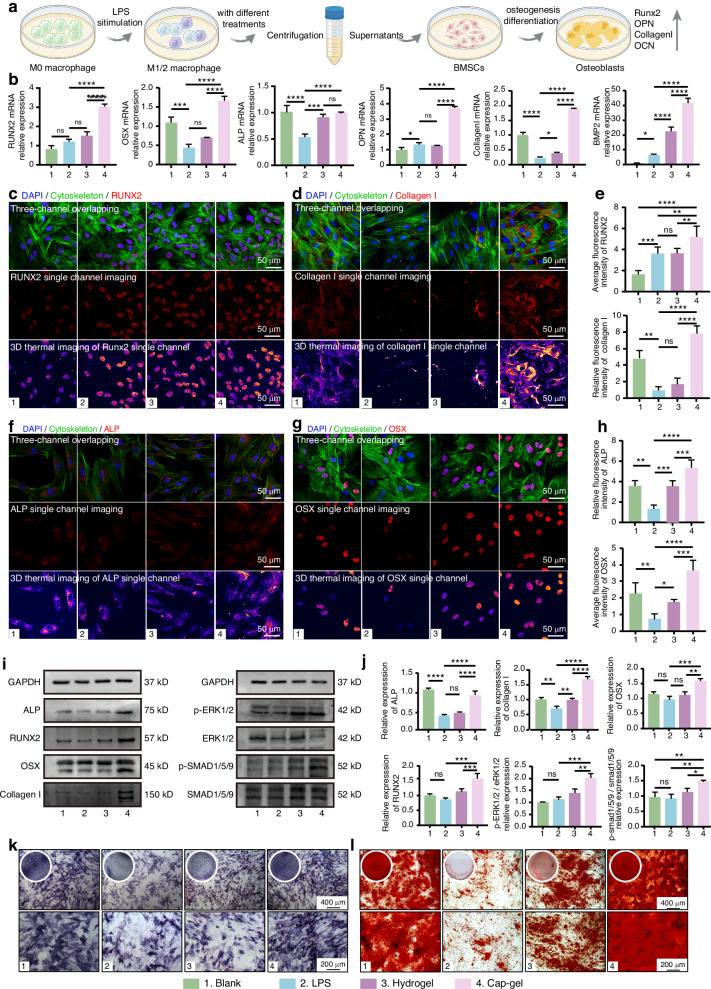
Mineralized tFNAs-based hydrogels indirectly regulate the osteogenic activity of BMSCs in vitro. **a** Schematic diagram of treating macrophages and BMSCs. **b** qPCR results of different treatment groups. Immunofluorescence detection images of RUNX2 (**c**) and Collagen I (**d**) in BMSCs (scale bar: 50 μm). **e** Statistical results of relative fluorescence intensity of different proteins in (**c**, **d**). Immunofluorescence detection images of ALP (**f**) and OSX in BMSCs (**g**) (scale bar: 50 μm). **h** Statistical results of relative fluorescence intensity of different proteins in (**f**, **g**). **i** Western blot results of various proteins in BMSCs. **j** Statistical analysis of the relative expression levels of various proteins normalized to GAPDH. **k** Alkaline phosphatase staining results after 14 days. **l** Alizarin red staining results after 14 days, scale bars: 400 μm (upper row), 200 μm (lower row). The complete image of the corresponding orifice plate is placed in the upper left corner. Data are presented as mean ± SD (*n* = 3), *P*-values are calculated using one-way ANOVA, *P < 0.05, **P < 0.01, ***P < 0.001, ****P < 0.000 1, ns no significance

Subsequently, we investigated the role of Cap-gel in the immune–osteogenic regulatory axis. To isolate its indirect effects mediated by macrophages, we first treated macrophages according to group assignment and collected their conditioned media, which were then applied to BMSCs (Fig. 4a). qPCR analysis revealed that inflammatory stimulation (LPS group) significantly suppressed the expression of key osteogenic genes such as OSX, ALP, and Collagen I in BMSCs compared to the Blank group (Fig. 4b). In contrast, conditioned media from Cap-gel-treated macrophages significantly restored and even enhanced the expression of these genes, indicating that Cap-gel effectively mitigates the negative impact of inflammation on osteogenic differentiation. Although Hydrogel treatment moderately upregulated certain markers (e.g., ALP, BMP2, Collagen I), its effects were consistently weaker than those of Cap-gel, likely due to its limited anti-inflammatory capacity and lack of osteoinductive mineral components.

Immunofluorescence imaging (Figs. 4c–h) validated these observations at the protein level. The upregulated expressions of ALP, OSX, RUNX2, OPN (Fig. S8), and Collagen I in the Cap-gel group were consistent with qPCR results. WB analysis further confirmed these trends (Fig. 4i): Cap-gel significantly upregulated ALP, RUNX2, OSX, and Collagen I, whereas LPS exposure alone reduced or failed to enhance their expression levels compared with the Blank group (Fig. 4j).

To elucidate the molecular mechanism by which Cap‑gel regulates the osteogenic differentiation of BMSCs, we examined the activation of key signaling pathways. WB results (Figs. 4i, j) showed that Cap-gel significantly activates the Smad pathway (evidenced by increased levels of p-Smad1/5/9) and induces downstream cascades (OCN, OPN, and Runx2), while specifically enhancing phosphorylation of the ERK-MAPK pathway (upregulation of p-ERK1/2). Since activation of Smad1/5/9 is commonly associated with elevated BMP2,^51^ and both Cap‑gel and Hydrogel induced macrophages to secrete comparable levels of BMP2, this commonality explains the activation of the BMP2/Smad pathway in both groups. However, the notable difference in ERK/MAPK activation between the two groups suggests the involvement of additional regulatory factors. A key distinguishing feature of Cap‑gel is its ability to sustainably release Ca^2+^. Literature widely reports that Ca^2+^ can independently activate proliferative and differentiation pathways such as ERK through the calcium-sensing receptor (CaSR) of BMSCs, enhancing the transcription of downstream osteogenic genes.^44–46^ Therefore, we propose that Ca^2+^ from Cap-gel not only serves as a mineralization substrate but also acts as a signaling molecule that specifically amplifies ERK/MAPK pathway signaling.^52^ Cap‑gel initiates BMP‑Smad signaling in BMSCs through macrophage‑secreted BMP2 and synergistically enhances the ERK/MAPK pathway via its released Ca^2+^, thereby establishing a dual‑signal cooperative network.

To assess functional outcomes, ALP staining and alizarin red staining were performed at the 7-day and 14-day time points. The Cap-gel group exhibited strong ALP activity and evident calcium nodule formation at day 7, comparable to the Blank group, whereas the LPS and Hydrogel groups showed fainter staining and negligible mineral deposition (Figs. S9–S11). By day 14, ALP activity increased across all groups, with the Cap-gel group exhibiting the most extensive and dense mineralized nodules. In contrast, the LPS and Hydrogel groups showed sparse and scattered mineral deposits (Figs. 4k, l, Figs. S9–S11).

Collectively, these findings demonstrated that Cap-gel could indirectly enhance BMSC osteogenesis by modulating macrophage polarization and suppressing pro-inflammatory responses, substantially promoting the expression of osteogenic markers and extracellular matrix mineralization and ultimately supporting bone regeneration.

### In vivo immune modulation and osteogenesis promotion by mineralized tFNAs-based hydrogels

**Fig. 5:**
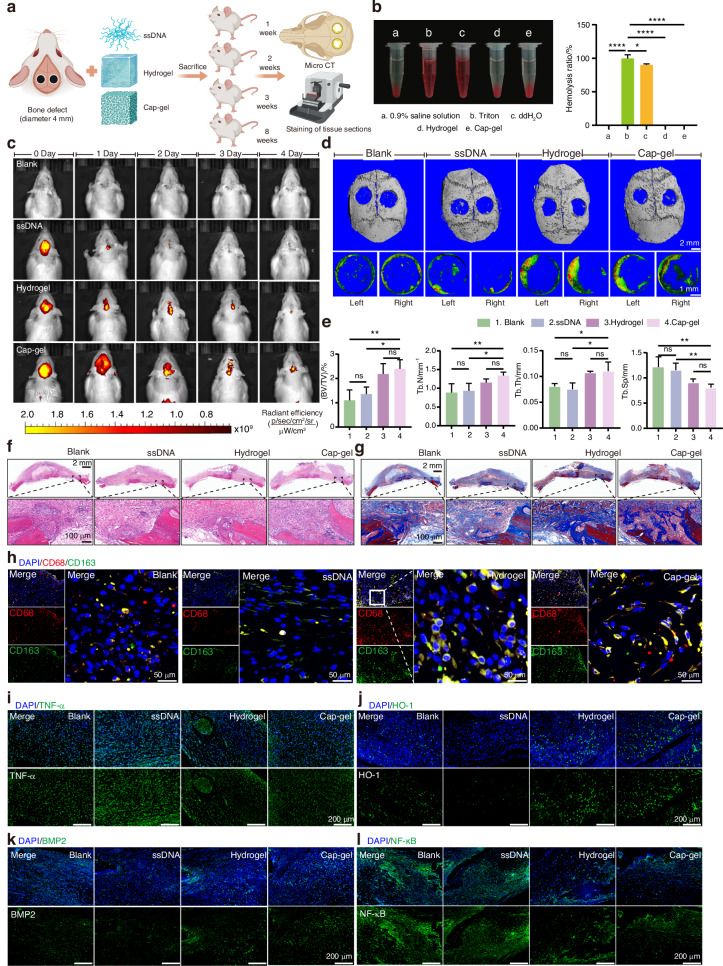
Mineralized tFNAs-based hydrogels short-term regulate the local immune status in vivo to promote osteogenesis. **a** Schematic diagram of the establishment of the animal model and in vivo experimental procedure. **b** Hemolysis results and hemolysis rate statistical results. **c** Live imaging of different filling materials in the rat cranial defect model. **d** Micro-CT reconstruction results of rat cranial defects at 1 week post-surgery in different treatment groups, scale bars: 2 mm (upper row), 1 mm (lower row). **e** Statistical results of Trabecular Number (Tb.N), Trabecular Separation (Tb.Sp), Trabecular Thickness (Tb.Th), Bone Volume over Total Volume (BV/TV). Data are presented as mean ± SD (*n* = 3), p-values are calculated using one-way ANOVA, *P < 0.05, **P < 0.01, ***P < 0.001, ****P < 0.000 1, ns: no significance. HE staining (**f**) and Masson staining (**g**) of rat cranial defects at 1 week after surgery in different treatment groups, scale bars: 100 μm (lower row). **h** Immunofluorescence staining of CD68^+^/CD163^+^ showed distinct macrophage polarization patterns among treatment groups, scale bar: 50 μm. Immunofluorescence staining of TNF-α (**i**), HO-1 (**j**), BMP2 (**k**) and NF-κB (**l**), scale bar: 200 μm

To further investigate the immunomodulatory and osteoinductive effects of Cap-gel in vivo, a rat critical-sized calvarial bone defect (CSBD) model was established to assess its influence on the local immune microenvironment and early bone regeneration (Fig. 5a). First, the safety performance of the material is evaluated. The venous blood of rats was collected, and normal saline, triton, ddH_2_O, Hydrogel and Cap-gel were added to observe the destruction of red blood cells and the hemolysis rate was counted. The results showed that the hemolysis rate of Hydrogel and Cap-gel was comparable to that of saline, i.e., no hemolysis occurred (Fig. 5b). In addition, histological examination of the major organs of rats at 8 weeks post-implantation showed no significant organ damage or inflammatory infiltration, demonstrating the safety of Cap-gel and hydrogel in vivo (Fig. S12). Then Cy5-labeled materials were tracked in vivo to determine their retention profiles. As shown in Fig. 5c and Fig. S13, non-crosslinked ssDNAs were rapidly degraded within 1 day, whereas both Hydrogel and Cap-gel persisted for at least 5 days. Notably, the Cap-gel group displayed a broader fluorescence distribution compared to the Hydrogel group, likely due to the stabilizing effect of calcium phosphate mineralization on the DNA framework.

Micro-CT analysis of calvarial samples collected at 1 week post-surgery showed negligible new bone formation in the Blank and ssDNA groups, while both Hydrogel and Cap-gel groups exhibited improved defect bridging and mineral deposition (Fig. 5d). Quantitative morphometric analysis (Fig. 5e) demonstrated that both Cap-gel and Hydrogel increased bone volume fraction (BV/TV), trabecular thickness (Tb.Th), and trabecular number (Tb.N) compared with Blank and ssDNA controls, indicating their ability to promote early osteogenesis. Although Cap-gel yielded slightly higher values than Hydrogel across all parameters, the differences were not statistically significant, suggesting comparable early-stage efficacy. This similarity may be attributed to their shared ability to modulate the immune response and improve the osteogenic microenvironment. However, the similar early outcomes highlight a key advantage of Cap-gel, which maintains excellent immunocompatibility while providing releasable calcium ions as a second, direct osteogenic cue. This dual action may enhance the momentum and efficiency of repair at later stages.

Histological analyses further corroborated these findings. Hematoxylin and Eosin (HE) staining (Fig. 5f) revealed distinct tissue responses among groups. The Blank and ssDNA groups exhibited extensive fibrous tissue infiltration and significant inflammatory cell accumulation. Hydrogel-treated defects showed limited bone-like tissue formation at the margins, while Cap-gel promoted denser tissue organization with evident osteoid formation and attenuated inflammation, indicating enhanced early-stage repair. Masson’s trichrome staining (Fig. 5g) showed minimal collagen deposition across all groups, with localized blue staining observed only in Hydrogel and Cap-gel groups at the defect edges, indicative of early collagen matrix formation. Notably, Cap-gel displayed slightly greater collagen deposition than Hydrogel. Immunofluorescence characterization (Fig. 5h) of macrophage polarization showed predominance of CD68^+^ M1 macrophages in Blank and ssDNA groups. Hydrogel treatment moderately increased CD163^+^ M2 macrophages, indicating partial immunomodulatory activity. Notably, Cap-gel induced a pronounced shift toward M2 polarization, characterized by abundant CD163^+^ cells and reduced CD68^+^ expression, thereby establishing a more pro-regenerative immune microenvironment.

To further evaluate the early immunomodulatory capacity and mechanism of the materials at the defect site, tissue sections were stained for TNF-α (a pro‑inflammatory factor) and HO‑1 (an anti‑inflammatory factor). The results showed decreased TNF‑α expression and reduced overall fluorescence intensity in both the Cap‑gel and Hydrogel groups (Fig. 5i), while HO‑1 expression was upregulated in these two groups (Fig. 5j). More importantly, a clear increase in BMP2 expression was observed in Cap‑gel‑treated tissue (Fig. 5k), providing key evidence that immunomodulation‑driven M2 macrophage polarization promotes a shift toward a reparative tissue response. Finally, the activation of the NF‑κB pathway—investigated in vitro—was examined in vivo. The results indicated that both Cap‑gel and Hydrogel suppressed NF‑κB expression (Fig. 5l). Thus, during the early stage of bone repair, Cap‑gel and Hydrogel primarily act by inhibiting the NF‑κB pathway, thereby attenuating local inflammation, reversing tissue damage, promoting the expression of key osteogenic proteins such as BMP2, and accelerating the transition of the defect site into a reparative phase.

**Fig. 6:**
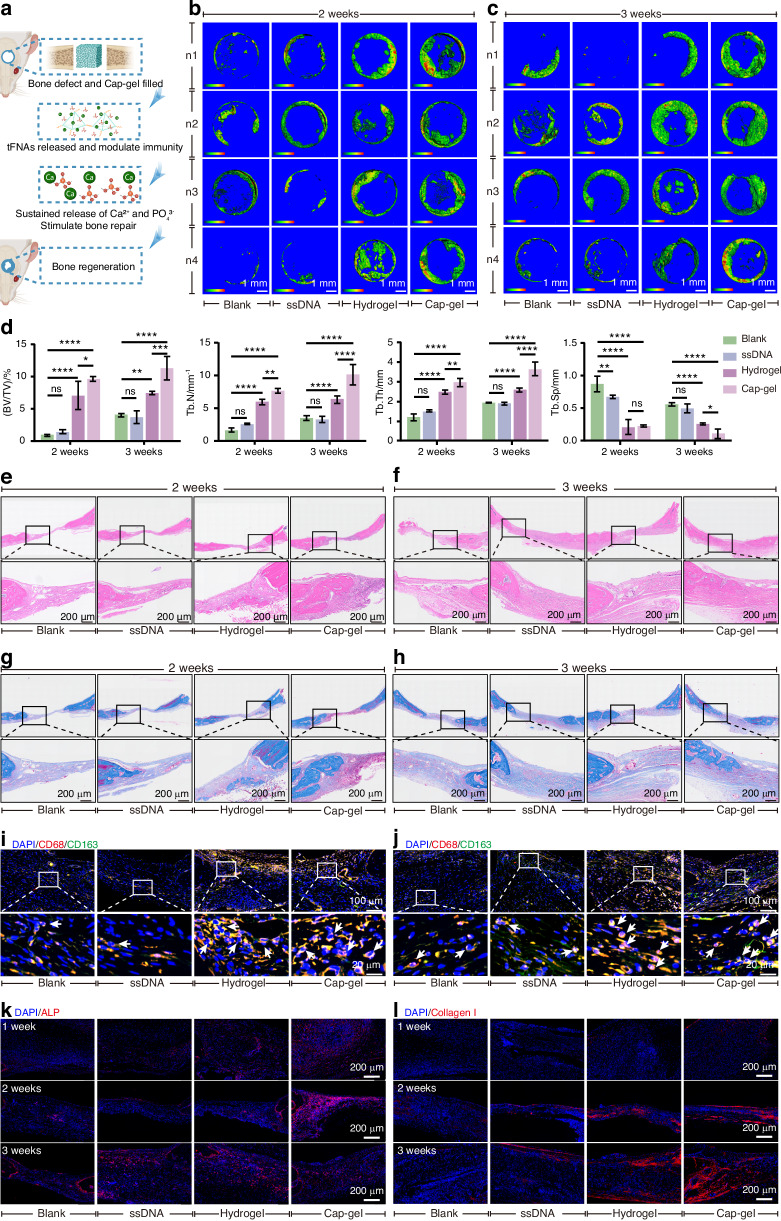
Mineralized tFNAs-based hydrogels exhibit sustained osteogenic capacity in sustainable evaluation. **a** Scheme of Cap-gel regulating osteogenic metabolism and local immunity. Micro-CT reconstruction results of rat cranial defects at 2 weeks (**b**, **c**) after surgery in different treatment groups, scale bar: 1 mm. **d** Statistical results of Trabecular Number (Tb.N), Trabecular Separation (Tb.Sp), Trabecular Thickness (Tb.Th), Bone Volume over Total Volume (BV/TV) in (b) and (**c**). Data are presented as mean ± SD (*n* = 4), *P*-values are calculated using one-way ANOVA, *P < 0.05, **P < 0.01, ***P < 0.001, ****P < 0.000 1, ns no significance. HE staining at 2 weeks (**e**) and 3 weeks (**f**) after surgery in different treatment groups, scale bar: 200 μm. Masson staining at 2 weeks (**g**) and 3 weeks (**h**) after surgery in different treatment groups, scale bar: 200 μm. Immunofluorescence staining of CD68^+^/CD163^+^ at 2 weeks (**i**) and 3 weeks (**j**) showed distinct macrophage polarization patterns among treatment groups, scale bar: 20 μm. Immunofluorescence staining of ALP (**k**) and Collagen I (**l**) at 1-3 weeks, scale bar: 200 μm

Considering the immunoregulatory and osteoinductive properties of Cap-gel, we propose a multiphase mechanism underlying its bone repair efficacy in vivo. In the early stage, Cap-gel induces a phenotypic shift in the local immune environment toward a pro-regenerative state, thus facilitating tissue regeneration. As the DNA nanostructures degrade, calcium and phosphate ions are gradually released, serving as essential mineral precursors for new bone formation. Meanwhile, Cap-gel directly and indirectly modulates BMSC behavior, enhancing osteogenic differentiation and matrix deposition (Fig. 6a). Micro-CT analysis at 2 and 3 weeks (Figs. 6b, c) revealed progressive bone regeneration in all groups, with minimal bone fill in Blank and ssDNA groups and more substantial new bone regeneration in Hydrogel and Cap-gel groups. Quantitative analysis (Fig. 6d) showed that Cap-gel yielded the highest BV/TV values (~7.5% at 2 weeks, ~10% at 3 weeks), along with significantly decreased Tb.Sp and increased Tb.N and Tb.Th, indicating enhanced trabecular microarchitecture and bridging. Remarkably, Cap-gel outperformed all groups by week 3, demonstrating superior mid-term bone repair potential.

Histological evaluation at 2 weeks (Figs. 6e, f) revealed distinct tissue responses. While control (Blank) and ssDNA groups displayed sparse fibrous tissue deposition and negligible bone regeneration, the Hydrogel-treated defects exhibited initial osteoid formation primarily at the peripheral regions. Notably, Cap-gel treatment resulted in more structurally defined bone matrix deposition, suggesting enhanced early reparative processes. By 3 weeks, both the Hydrogel and Cap-gel groups showed denser tissue and smaller defect gaps, with the Cap-gel group forming more continuous plate-like bone structures, suggesting advanced maturation. Masson’s trichrome staining (Figs. 6g, h) provided complementary evidence of early collagen and extracellular matrix deposition. At 2 weeks, only Hydrogel and Cap-gel treated defects exhibited faint blue staining at the peripheral regions. The Cap-gel group also showed predominant red staining in central defect areas, indicating the presence of unmineralized matrix and early tissue. By 3 weeks, extensive blue staining, particularly at bridging zones, was observed in the Cap-gel group, suggesting enhanced collagen deposition and bone formation.

Immunofluorescence staining of macrophage polarization (Figs. 6i, j) revealed significantly higher infiltration of CD163^+^ M2 macrophages in both Hydrogel and Cap-gel groups compared to Blank and ssDNA controls at all examined timepoints. Remarkably, Cap-gel treatment resulted in the most pronounced M2 macrophage accumulation, suggesting superior immunomodulatory effects. Moreover, expression levels of ALP (Fig. 6k), Collagen I (Fig. 6l), and OCN (Fig. S14) were markedly elevated and spatially continuous in the Cap-gel group, indicating robust osteogenic activity and effective bone matrix regeneration.

During the early to mid‑phase of bone repair, M2 macrophages induced by Cap‑gel predominated in the defect site. This not only alleviated local inflammatory infiltration but also, through the sustained release of calcium ions, promoted the upregulation of osteogenic regulatory proteins and accelerated the bone regeneration process. Considering that bone repair is a long‑term process, we extended the observation period to 8 weeks post‑operation to evaluate the final osteogenic outcome of Cap‑gel.

**Fig. 7:**
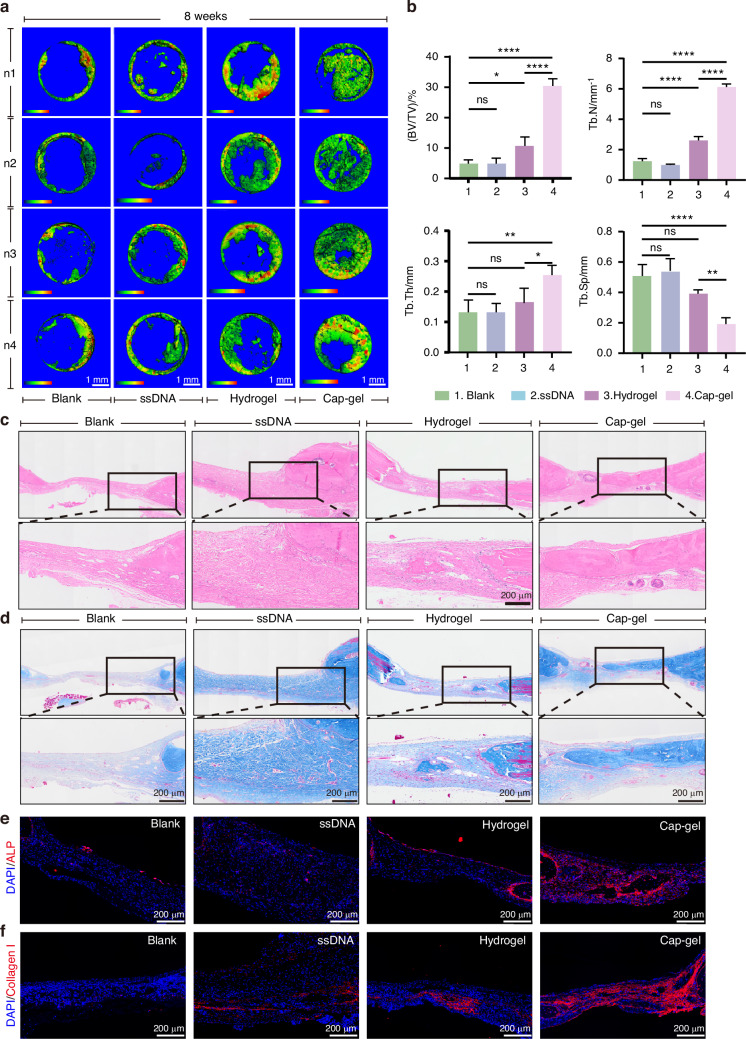
Mineralized tFNAs-based hydrogels exhibit excellent long-term osteogenic effects. **a** Micro-CT reconstruction results of rat cranial defects at 8 weeks after surgery in different treatment groups, scale bar: 1 mm. **b** Statistical results of Trabecular Number (Tb.N), Trabecular Separation (Tb.Sp), Trabecular Thickness (Tb.Th), Bone Volume over Total Volume (BV/TV) in (**a**). Data are presented as mean ± SD (*n* = 4), *P*-values are calculated using one-way ANOVA, *P < 0.05, **P < 0.01, ***P < 0.001, ****P < 0.000 1, ns: no significance. HE staining (**c**) and Masson staining (**d**) at 8 weeks after surgery in different treatment groups, scale bar: 200 μm. Immunofluorescence staining of ALP (**e**) and Collagen I (**f**) at 8 weeks, scale bar: 200 μm

Micro‑CT results (Figs. 7a, b) showed markedly enhanced new bone formation in the Cap‑gel group after 8 weeks, whereas the Hydrogel group exhibited a lack of sustained efficacy with no significant increase in bone formation compared to the 3‑week time point. As expected, the Cap‑gel group demonstrated superior outcomes across all bone parameters. The bone volume fraction (BV/TV) reached (30.32 ± 5.79)%, significantly higher than that of other groups. Trabecular number and thickness were also notably increased. H&E staining (Fig. 7c) revealed continuous, plate‑like bone tissue in the Cap‑gel group, with abundant osteoblasts lining the surface of the newly formed bone matrix and the presence of a small number of multinucleated giant cells, indicating normal bone remodeling during the formation process. In contrast, the defect areas in the Blank and ssDNA groups remained filled with fibrous tissue. Masson’s trichrome staining (Fig. 7d) displayed deeper and more extensive blue staining in the Cap‑gel group compared to the Hydrogel group, suggesting higher mineralization and more mature bone formation.

Further detection of ALP (Fig. 7e) and Collagen I (Fig. 7f) expression showed strong positive signals in the Cap‑gel group, while only weak or scattered signals were observed in other groups. These findings suggest that the sustained release of calcium ions Cap‑gel can locally maintain osteoblast activity even at the late stage of repair, continuously driving bone matrix and collagen deposition.

## Discussion

This study addresses two main limitations of traditional DNA hydrogels in bone regeneration: the lack of active regulation of the osteoimmune microenvironment, and the inability to coordinate both immunomodulation and osteogenesis during repair. To overcome these, we developed a mineralized DNA hydrogel based on tFNAs, termed Cap-gel. Using tFNA nanoscaffolds as a template, controlled deposition and stable assembly of calcium phosphate nanocrystals were achieved. Multiple characterization results confirm the successful formation of a crystalline calcium phosphate mineral phase within Cap-gel, which enables uniform and sustained release of calcium ions while simultaneously improving the mechanical properties of the hydrogel while simultaneously improving the mechanical properties of the hydrogel, thereby enhancing its structural stability as a provisional matrix during the early healing phase.

The key advantage of Cap-gel lies in its spatiotemporally coupled dual functions. In the early stage of bone repair, it suppresses NF‑κB pathway activation, promotes macrophage polarization toward the M2 phenotype, mitigates oxidative stress, and shifts the local immune microenvironment toward a pro‑healing state. This process also induces macrophages to secrete BMP2. However, this study does not provide a complete macrophage transcriptomic profile. We only confirmed that Cap-gel modulates key pathways involved in inflammation suppression. Future, broader transcriptome sequencing may better clarify its mechanism of action. In the mid‑to‑late stages, Cap‑gel acts as a bioactive mineral reservoir, gradually releasing calcium during degradation to support bone matrix deposition. These ions, together with BMP2 and other pro‑healing factors secreted earlier, synergistically activate the ERK/MAPK and BMP/Smad pathways in BMSCs, thereby efficiently promoting osteogenic differentiation and mineralization.

This coupled mechanism of osteoimmune regulation was validated using an indirect macrophage‑BMSC co‑culture system and a rat calvarial defect model observed at multiple time points (1, 2, 3, and 8 weeks). The multi‑week observation window allowed us to capture the material’s functional transition from early immunomodulation to accelerated osteogenesis and, finally, to enhanced defect healing. It should be noted that the rat calvarial defect model, while widely used for preliminary evaluation of bone‑regeneration materials, is non‑load‑bearing and cannot replicate the complex biomechanical stresses present in load‑bearing bone repair. The performance of Cap‑gel in load‑bearing defects remains an important direction for future research.

In summary, Cap‑gel not only offers a new strategy for applying DNA biomaterials in bone tissue engineering, but also demonstrates the feasibility of achieving combined immunomodulation and osteogenic repair through nanostructural design. It holds significant translational potential in craniofacial bone repair, complex trauma regeneration, and chronic bone healing disorders. Future studies should focus on its long‑term safety and efficacy validation in large animal models to facilitate clinical translation.

## Materials and methods

### Preparation of mineralized tFNAs-based hydrogels

#### Synthesis of basic tFNAs structural units

TFNAs were constructed by combining four equimolar ssDNA strands (S1-S4). The termini of each ssDNA form the four vertices of the tFNA. Complementary sticky ends were introduced through 5’ terminal modifications using either sequence “a” (S1a-S4a) or its reverse complement “a*” (S1a*–S4a*), forming tFNA-a (Ta) and tFNA-a* (Ta*), respectively. Sequences are listed in Table S1. Lyophilized ssDNAs (Sangon Biotech, Shanghai) were dissolved in nuclease-free water. The four ssDNA strands were combined at 100 μmol/L stoichiometry in TM buffer (10 mmol/L Tris-HCl, 10 mmol/L MgCl₂, 1 mmol/L EDTA, pH 8.0) for equimolar assembly. Following mixing and centrifugation, the sample underwent thermal cycling (95 °C, 10 min) with subsequent rapid cooling to 4 °C (20 min). Samples were purified by 30 kD ultrafiltration to remove excess ssDNA and stored at 4 °C.

#### Synthesis of TFNAs-based nucleic acid hydrogel

TFNAs-based nucleic acid hydrogel (called Hydrogel) was prepared by mixing Ta and Ta* (1:1 molar ratio) under isothermal conditions and incubated directly at 65 °C. The resulting hydrogel was then stored at low temperature to maintain its structural stability.

#### Synthesis of mineralized TFNAs-based hydrogel based on calcium phosphate crystals (Cap-gel)

At room temperature (25 °C), the tFNAs-based hydrogel was loaded into cellulose dialysis membranes (MWCO 100–500 Da; Yuanye, Shanghai, China). First, 300 mmol/L K₂HPO₄ solution was continuously dropped outside the dialysis menbranes for 60 s, followed by rinsing with double-distilled water (ddH₂O) for 60 s. Then, 500 mmol/L CaCl₂ solution was dropped in the same manner for 60 s and again rinsed with ddH₂O for 60 s, completing one mineralization cycle. This cycle was repeated five times. The hydrogel was then extracted and transferred to an Eppendorf tube, immersed in 500 mmol/L CaCl₂ solution, and incubated without agitation at 37 °C for 4 h to obtain the final Cap-gel.

### Microstructural characterization of hydrogel and Cap-gel

After synthesizing the tFNAs-based hydrogel (Hydrogel) and its mineralized counterpart (Cap-gel), the samples were dehydrated at low temperature and freeze-dried using a vacuum lyophilizer. Freeze-dried hydrogel samples were sectioned, gold-sputtered, and analyzed via SEM to characterize their microstructures in the dry state and evaluate the pore size distribution and mineralization homogeneity.

### Crystal structure and elemental analysis of Cap-gel

#### HRTEM

The internal structure of the Cap-gel was examined using HRTEM to verify the formation and deposition of calcium phosphate crystals and to observe their lattice spacing. Ultrathin sections (thickness <100 nm) of the hydrogel were placed on copper grids and observed using HRTEM (JEOL JEM-2100F), with an accelerating voltage of 200 kV. Lattice fringe images were acquired in high-resolution imaging mode, and the crystal spacing was measured using image analysis software (Gatan DigitalMicrograph).

#### Fast Fourier Transform (FFT)

In HRTEM imaging mode, specific regions of the Cap-gel were selected to obtain high-resolution lattice images. FFT was applied to convert the images from real space to reciprocal space, generating diffraction spot patterns.

#### HAADF-STEM

Ultrathin sections of the Cap-gel were placed on copper grids and examined using HAADF-STEM, e.g., FEI Titan Themis, operated at 200 kV. In combination with EDS, characteristic X-ray emissions of elements including C, N, O, P, and Ca were collected. Elemental mapping was performed to visualize the spatial distribution of these elements.

#### FTIR

Samples were prepared using the potassium bromide (KBr) pellet method. Hydrogel, Cap-gel, CaCl₂, Na₂HPO₄, and standard calcium phosphate (Sigma, USA) were each mixed with dried KBr at a mass ratio of 1:100, homogeneously ground, and pressed into transparent pellets. FTIR spectra (Nicolet iS50) were acquired from 4 000 to 400 cm^−1^ (4 cm^−1^ resolution, 32 scans) in transmission mode. A pure KBr pellet was used for background correction to eliminate environmental interference. Spectra of the five samples were then collected, and baseline correction and peak calibration were conducted using OMNIC software.

#### XRD

The crystalline structures of Hydrogel and Cap-gel were analyzed using an X-ray diffractometer (Rigaku Ultima IV, Bruker D8 Advance). Freeze-dried samples were ground into fine powders and flattened into the sample holder for measurement. XRD analysis used Cu Kα radiation (λ = 1.540 6 Å) at 40 kV/40 mA, scanning 10°–90° 2θ (0.02° step, 5°/min). The resulting diffraction patterns were processed using Jade 6 software for baseline correction and smoothing. The crystalline structure of the mineralized product was identified by comparing the diffraction peaks with the brushite calcium phosphate reference (PDF#73-0293).

#### XPS

Elemental composition and chemical state analyses of the Hydrogel and Cap-gel were performed using an X-ray photoelectron spectrometer (Thermo Scientific K-Alpha). Freeze-dried samples were mounted on the specimen stage and analyzed under ultrahigh vacuum conditions (<10⁻⁸ mbar). XPS analysis employed Al Kα radiation (1 486.6 eV) at 50 eV pass energy and 0.1 eV step size. A survey scan was first conducted to identify the elements present (e.g., C, N, O, P, and Ca) and their relative abundances. Element-specific scans (C 1 s, N 1 s, O 1 s, P 2p, Ca 2p) were performed at higher resolution (20 eV pass, 0.05 eV step). Peak fitting and deconvolution of the spectra were carried out using Avantage software.

### Animals

Two-week-old and six-week-old male Sprague-Dawley (SD) rats purchased from Ensiweier (Chongqing, China) were used in the experiments. The study was approved by the Ethics Committee of Sichuan University (approval number: WCHSIRB-D-2-23-608).

### Isolation of rat BMSCs

Two-week-old male SD rats were euthanized and sterilized with 75% ethanol, and transferred to a biosafety cabinet. Femurs and tibias were isolated, immersed in PBS, and the epiphyses removed. Bone marrow was flushed out with complete culture medium (MEM-α supplemented with 10% FBS and 1% penicillin/streptomycin) using a 5 mL syringe, repeated 3-5 times until the effluent was clear. The suspension was mixed and transferred to T25 flasks for culture. Medium was half-changed every other day, and fully refreshed on day 3. Passage 3 (P3) BMSCs (confirmed by CD90^+^/CD45^−^ expression) were used for all experiments. For seeding, cells were plated onto gelatin-coated 12-well plates at 6 × 10⁴ cells/mL and incubated overnight.

#### Regulation of macrophage inflammation by Cap-gel

RAW264.7 cells were allocated to four experimental groups: Blank, LPS, Hydrogel, and Cap-gel. All groups were serum-starved for 6 hours, then treated for 3 h with 1 μg/mL LPS (except the Blank group). After removing the medium, cells were cultured for 24 h in high-glucose DMEM with 2% FBS (Blank and LPS groups), non-mineralized tFNAs-based hydrogel extract (Hydrogel group), or mineralized tFNAs-based hydrogel extract (Cap-gel group), both at a 1:20 dilution in medium. Cells were then collected for analysis.

#### Regulation of BMSC osteogenesis by Cap-gel

BMSCs were divided into four groups: Blank, ssDNA, Hydrogel, and Cap-gel. After 6 h of serum starvation, cells were treated as follows: the Blank group received complete MEM-α with 10% FBS; the ssDNA group received ssDNA at a concentration equivalent to that in the tFNAs-based hydrogel extract; the Hydrogel and Cap-gel groups received tFNAs-based hydrogel and mineralized tFNAs-based hydrogel extracts (1:20 dilution in complete medium), respectively. All groups were supplemented with osteogenic inducers (OriCell, RAXMX-90021, China). Culture medium was refreshed every 3 days, with cells harvested at 7- and 14-day timepoints for analysis.

#### Indirect regulation of BMSC osteogenesis via inflammatory macrophage secretions

RAW264.7 cells were divided into groups and treated as described in Section “Regulation of Macrophage Inflammation by Cap-ge”. After 24 h, supernatants were collected and centrifuged (1 000 r/min, 5 min). The supernatants were used to treat BMSCs seeded in another plate. Osteogenic inducers were added simultaneously as described in “Regulation of BMSC Osteogenesis by Cap-gel”. BMSCs were collected on days 7 and 14 for subsequent assays.

### Animal model

Male SD rats (6-week-old) underwent anesthesia with 2% isoflurane (induction) and 1.5% (maintenance), combined with local 2% lidocaine (0.1 mL/site). A 2 cm midline scalp incision was made to expose the calvaria. Bilateral 4 mm full-thickness critical-sized defects were created along the sagittal suture using a trephine drill with continuous saline irrigation. The dura mater was carefully preserved during defect creation. Animals were randomly allocated to four treatment groups (*n* = 6): Blank, ssDNA, Hydrogel, or Cap-gel (50 μL each), with subsequent collagen membrane coverage. The incision was sutured in layers using 5–0 absorbable sutures. Penicillin was injected intraperitoneally for 3 days post-op. At 7-/14-/21-day endpoints, calvarial specimens (defect area) were harvested after euthanasia, fixed in 4% paraformaldehyde, and PBS-preserved for multimodal analysis.

### Statistical analysis

The statistical analysis of all experimental data was performed using GraphPad Prism software, version 9.0.0 (San Diego, USA). The comparison between groups involved one-way analysis of variance (ANOVA) as a statistical method. The means ± standard deviations (SD) (*n* ≥ 3) were used to present quantitative data.

## Supplementary information

SUPPLEMENTAL MATERIAL

## Data Availability

The data that support the findings of this study are available from the corresponding author upon reasonable request.
