# Supplementary material for: Mineralized DNA tetrahedron-structured hydrogels: a dual-functional Scaffold for immunomodulation and bone regeneration
Source: Bone Res. 2026 May 8;14:50. doi: 10.1038/s41413-026-00530-8 (PMC13156309; doi:10.1038/s41413-026-00530-8)
Supplement: Supplementary file 1 — SUPPLEMENTAL MATERIAL [file 41413_2026_530_MOESM1_ESM.docx]

**Supporting Information**

Mineralized DNA Tetrahedron-Structured Hydrogels: A Dual-Functional Scaffold for Immunomodulation and Bone Regeneration

Lan Yao^a,1^, Jiafei Sun^c,1^, Zhiqiang Liu^a^, Jiale Liang^a^, Yun Wang^a^, Ye Chen^a^, Ruiqing Wang^a^, Tao He^a^, Yichen Yang^a^, Yao He^d^, Yunfeng Lin^a,b,*^, Taoran Tian ^a,b,*^

a

State Key Laboratory of Oral Diseases, National Center for Stomatology, National Clinical Research Center for Oral Diseases, West China Hospital of Stomatology,

Sichuan University, Chengdu, Sichuan, 610041, China

b

Sichuan Provincial Engineering Research Center of Oral Biomaterials, Chengdu, Sichuan, 610041, China

c

Stomatology Hospital, School of Stomatology, Zhejiang University School of Medicine, Zhejiang Provincial Clinical Research Center for Oral Diseases, Key Laboratory of Oral Biomedical Research of Zhejiang Province, Cancer Center of Zhejiang University, Engineering Research Center of Oral Biomaterials and Devices of Zhejiang Province, Hangzhou, 310000, China

d

Macao Translational Medicine Center, Macau University of Science and Technology. Taipa 999078, Macau SAR, China，

^*^Correspondence:

Yunfeng Lin (yunfenglin@scu.edu.cn)

Taoran Tian ( [taoran.tian@scu.edu.cn](mailto:taoran.tian@scu.edu.cn))

Materials and Methods

Rheological and Mechanical Characterization of Cap-gel

Y-type hydrogel (Y-Hydro) was synthesized following a previous method^1, 2^, using the same number of bases as Hydrogel. Specifically, Ys1, Ys2, and Ys3 DNA strands were prepared into Y-scaffold at 1× concentration, and Ls1 and Ls2 DNA strands were synthesized into Linker at 1.5× concentration (sequence reference table S1). Equal volumes of Y-scaffold and Linker were mixed to obtain Y-Hydro. Hydrogel and Cap-gel were synthesized using the same method. Rheological properties were evaluated using a stress-controlled rheometer (MCR302, Anton Paar, Austria). A 5-minute dynamic oscillatory time sweep test was conducted to assess the gel formation (strain: 1%, frequency: 1 Hz, 25°C). Mechanical properties were evaluated using a universal testing machine (UTM4103, Shenzhen, China).

Ion Release Test

Cap-gel was synthesized as described above, placed in a 30 kDa ultrafiltration membrane, sealed, and placed in a 50 mL centrifuge tube. Fifty times the volume of external solution was added, and the mixture was incubated at room temperature with continuous shaking for 1, 3, 5, 7, 14, and 21 days. The external solution was then collected and analyzed for calcium ion concentration using an inductively coupled plasma optical emission spectrometer (PE Optima 8000, PerkinElmer, USA).

In vitro DNA Degradation Test

Hydrogel and Cap-gel, synthesized as described above, were placed in a 30 kDa ultrafiltration membrane, sealed, and placed in a 50 mL centrifuge tube. Fifty times the volume of simulated body fluid (SBF) containing 5 U/mL DNase I was added, and the mixture was incubated at room temperature with continuous shaking for 6, 9, 12, 24, 48, 72, 120, and 168 hours. The external solution was collected and DNA concentration was measured using a UV spectrophotometer.

ROS Detection

Intracellular ROS levels were determined using DCFH-DA probe (Beyotime kit, China). After 24 hours of treatment in 12-well plates, the culture medium was removed, and cells were incubated with 10 μM DCFH-DA (37°C, 20min, dark). Following PBS washes, ROS was visualized by fluorescence microscopy and quantified with ImageJ.

ALP and Alizarin red staining

After 7 or 14 days of osteogenic induction, cells were washed twice with PBS and fixed in 4% (w/v) paraformaldehyde for 10 min. For ALP staining, fixed cells were incubated with BCIP/NBT staining solution (Beyotime, China) according to the manufacturer's instructions, followed by three rinses with ddH₂O. For calcium deposition assessment, cells were stained with Alizarin Red solution (OriCell, RAXMX-90021, China) after ddH₂O rinsing. Stained regions (blue-purple for ALP, red for calcium nodules) were observed and imaged under a microscope. Furthermore, the mineralized nodules formed under the culture conditions of each group were semi-quantitatively analyzed using Cetylpyridinium chloride (CPC, Solarbio, China). To be brief, the stained calcium nodules described above were treated with 10 % CPC (w/v) for 15 min, and the absorbance at 562 nm was determined, which was quantified through comparing to the standard curve of calcium concentration.

Micro CT Analysis

Rat skulls were scanned using a Micro CT system (TD-273 Micro CT 50/100 version 1.3, SCANCO MEDICAL AG, Zurich, Switzerland) with a resolution set at 10 μm for 3D image reconstruction. Region of interest (ROI) mapping on 2D slices and subsequent 3D reconstructions were performed using identical threshold values across all groups. Quantitative analysis of the Micro CT data was conducted using SCANCO evaluation software to assess cranial defect repair and new bone formation at 1, 2, 3 and 8 weeks post-treatment. Parameters analyzed included 3D reconstruction of the skull and measurements of bone volume fraction (BV/TV, Tb.N, Tb.Th, and Tb.Sp).

Histological and IF Evaluation

Following Micro CT scanning, bone samples were decalcified in ethylene diamine tetraacetic acid (EDTA, 0.5 mol/L, pH 7.2) solution. The decalcification solution was refreshed every 3 days until complete decalcification was achieved, as determined by needle-puncture test in which a pin could easily penetrate the bone tissue (approximately 4 weeks, based on the preliminary experiments). The decalcified samples were then embedded in paraffin, sectioned, and subjected to hematoxylin and eosin (H&E) staining, Masson's trichrome staining, and IF staining. The results were observed using a light microscope and slide scanner. H&E and Masson staining were used to evaluate the volume fraction of newly formed collagen fibers, new bone formation extent, and regenerated tissue maturity across different groups. For IF staining, sections were incubated overnight at 4°C with primary antibodies against ALP (ET1601-21, Huabio), OCN (ER1919-20, Huabio), Collagen I (HA722517, Huabio), NF-κB p65 (8242, CST), HO-1 (HA721854, Huabio), BMP2 (ER80602, Huabio), TNF-α (ab215188, Abcam), CD68 (ab283654, Abcam), and CD163 (ab316218, Abcam), followed by secondary antibodies. Nuclei were counterstained with DAPI. Stained sections were imaged using a microscope and slide scanner. Quantitative analysis of immunofluorescence signals was performed with ImageJ, and data were compared among groups to determine the anti-inflammatory effects and osteogenic repair capacity of the Cap-gel in the rat cranial defect model.

qPCR

After 24 hours of culture according to the experimental grouping, total RNA was extracted. One microgram of RNA was used for reverse transcription. The resulting cDNA was used for qPCR with primers targeting ALP, OSX, OPN, Collagen I, RUNX2, BMP2, and the internal reference gene GAPDH (sequences listed in Table 2). The cycling protocol was: 95 °C for 30 s, followed by 40 cycles of 95 °C for 5 s and 60 °C for 34 s. Relative gene expression was calculated using the 2⁻^ΔΔCt method with GAPDH as internal control.

WB

Total protein was extracted using a commercial extraction kit (KGB5303, KeyGEN) per the manufacturer’s protocol. Protein samples were separated by SDS-PAGE and transferred to PVDF membranes using a semi-dry blotting system. Membranes were blocked with blocking buffer (Beyotime, China) for 10 minutes, followed by incubation with primary antibodies at 4 °C overnight. The primary antibodies used included: GAPDH (1:1000, 5174S, CST), TNF-α (1:1000, ab215188, Abcam), IL-6 (1:1000, ab290735, Abcam), iNOS (1:1000, ab178945, Abcam), IL-10 (1:1000, ER1911-19, Huabio), NF-κB p65 (1:1000, ab16502, Abcam), phospho-p65 (1:1000, 3033, CST), HO-1 (1:1000, HA721854, Huabio), BMP2 (1:1000, ER80602, Huabio), OSX (1:1000, HA722817, Huabio), Osteopontin (OPN, 1:1000, 0806-6, Huabio), Collagen I (1:1000, HA722517, Huabio), Runx2 (1:1000, ET1612-47, Huabio), ALP (1:1000, ET1601-21, Huabio), ERK1/2 (1:1000, 4695, CST), phospho-ERK1/2 (1:1000, 4370, CST), SMAD1/5/9(1:1000, ab300164, Abcam), phospho-SMAD1/5/9 (1:5000, ab92698, Abcam). After equilibration (30 min, RT) and TBST washes, membranes were incubated with HRP-conjugated secondary antibody (1:3000, Beyotime) for an hour. After washing, protein bands were visualized using chemiluminescence reagents and imaged using the iBright FL1500 system.

IF

After 24 hous treatment, cells were washed and subjected to fixation, permeabilization, and blocking procedures, followed by overnight incubation at 4°C with primary antibodies: TNF-α (1:500, ab215188, Abcam), IL-6 (1:200, ab290735, Abcam), iNOS (1:500, ab178945, Abcam), HO-1 (1:200, HA721854, Huabio), OSX (1:200, HA722817, Huabio), Osteopontin (OPN, 1:200, 0806-6, Huabio), Collagen I (1:200, HA722517, Huabio), Runx2 (1:200, ET1612-47, Huabio), and ALP (1:500, ET1601-21, Huabio). The next day, after PBS washing and 30 min rewarming, cells were incubated with A594-conjugated secondary antibodies at 37 °C for one hour. Rhodamine-phalloidin and DAPI were applied sequentially for cytoskeleton and nuclear staining. Samples were mounted with 10% glycerol and observed under a confocal microscope.

Flow Cytometry

RAW264.7 cells were harvested after treatment. Fc receptors were blocked with anti-CD16/CD32 antibodies (BioLegend), followed by staining at 4 °C for 30 min with fluorescently labeled monoclonal antibodies according to the experimental design. For intracellular markers, cells were processed using a Fixation/Permeabilization Solution Kit with BD GolgiPlug (BD Biosciences), then stained with specific antibodies for 30 min at room temperature. The antibodies used included: APC-conjugated anti-CD80 (BioLegend), PE-conjugated anti-CD206 (BioLegend). Data were acquired using an Attune NxT flow cytometer and analyzed with FlowJo10.6.2 software (Treestar, Ashland, Ore).

Hemolysis test

Rat abdominal vein blood with added sodium citrate was diluted 1:1 with normal saline, with 100 uL added to each 1.5ml centrifuge tube. To tubes a, b, c, d, and e, 1 mL of 0.9% normal saline, 10% Triton, deionized water, 10% Hydroge, and 10% cap-gel were added respectively. After standing at 37°C for 30 min, the samples were centrifuged at 1500 rpm for 5 min. The OD value of the supernatant was measured at 545 nm. The hemolysis rate of the material was calculated using the following formula:

Hemolysis rate (%) = [(OD sample - OD negative control) / (OD positive control - OD negative control)] × 100%

In Vivo Living Image Analysis

ssDNA, Hydrogel, or Cap-gel labeled with Cy5 fluorophores was applied to rat CSBDs. At designated time points (days 0, 1, 2, 3, and 5), fluorescence intensity and distribution at the defect site were monitored using an in vivo imaging system (IVIS Lumina III, USA).

**Table S1.** Sequences of every ssDNAs with sticky ends.

| Strand Name | Base Sequence (from 5' to 3', Sticky End in Bold) |
| --- | --- |
| S1a | ATTTATCACCCGCCATAGTAGACGTATCACCAGGCAGTTGAGACGAACATTCCTAAGTCTGAATT**TTCCTCTACCACCTACATCAC** |
| S2a | ACATGCGAGGGTCCAATACCGACGATTACAGCTTGCTACACGATTCAGACTTAGGAATGTTCGTT**TTCCTCTACCACCTACATCAC** |
| S3a | ACTACTATGGCGGGTGATAAAACGTGTAGCAAGCTGTAATCGACGGGAAGAGCATGCCCATCCTT**TTCCTCTACCACCTACATCAC** |
| S4a | ACGGTATTGGACCCTCGCATGACTCAACTGCCTGGTGATACGAGGATGGGCATGCTCTTCCCGTT**TTCCTCTACCACCTACATCAC** |
| S1a* | ATTTATCACCCGCCATAGTAGACGTATCACCAGGCAGTTGAGACGAACATTCCTAAGTCTGAATT**GTGATGTAGGTGGTAGAGGAA** |
| S2a* | ACATGCGAGGGTCCAATACCGACGATTACAGCTTGCTACACGATTCAGACTTAGGAATGTTCGTT**GTGATGTAGGTGGTAGAGGAA** |
| S3a* | ACTACTATGGCGGGTGATAAAACGTGTAGCAAGCTGTAATCGACGGGAAGAGCATGCCCATCCTT**GTGATGTAGGTGGTAGAGGAA** |
| S4a* | ACGGTATTGGACCCTCGCATGACTCAACTGCCTGGTGATACGAGGATGGGCATGCTCTTCCCGTT**GTGATGTAGGTGGTAGAGGAA** |
| Ys1 | CGATTGACTCTCCACGCTGTCCTAACCATGACCGTCGAAG |
| Ys2 | CGATTGACTCTCCTTCGACGGTCATGTACTAGATCAGAGG |
| Ys3 | CGATTGACTCTCCCTCTGATCTAGTAGTTAGGACAGCGTG |
| Ls1 | GAGAGTCAATCGTCTATTCGCATGAGAATTCCATTCACCGTAAG |
| Ls2 | GAGAGTCAATCGCTTACGGTGAATGGAATTCTCATGCGAATAGA |

| Target Gene | Primer pairs (5′ → 3′) |
| --- | --- |
| Mouse-GAPDH | Forward AGGTCGGTGTGAACGGATTTG Reverse TGTAGACCATGTAGTTGAGGTCA |
| Mouse-IL-6 | Forward TAGTCCTTCCTACCCCAATTTCC  Reverse TTGGTCCTTAGCCACTCCTTC |
| Mouse-TNF-α | Forward CCCTCACACTCAGATCATCTTCT  Reverse GCTACGACGTGGGCTACAG |
| Mouse-iNOS | Forward TCAGGAGGGCCTAATGAGCT  Reverse TTTGTACGCGGCTTCCTCTT |
| Mouse-IL-10 | Forward GCAGCCTTGCAGAAAAGAGA  Reverse CTGGGAAGTGGGTGCAGTTA |
| Mouse-BMP2 | Forward CCAGTCTTGCCGCCTCCAG  Reverse TCGCCTCCTCCTCCTTCTCC |
| Mouse-TGF-β | Forward GACCTGGGTTGGAAGTGGAT  Reverse TTGGTTGTAGAGGGCAAGGA |
| Rat-GAPDH | Forward GTCCATGCCATCACTGCCACTC  Reverse GATGACCTTGCCCACAGCCTTG |
| Rat-Runx2 | Forward GAACCAAGAAGGCACAGAC  Reverse AATGCGCCCTAAATCACTG |
| Rat-OPN | Forward CCAGCCAAGGACCAACTAC  Reverse AGTGTTTGCTGTAATGCGCC |
| Rat-OSX | Forward GGAGGCACAAAGAAGCCATA  Reverse GGGAAAGGGTGGGTAGTCAT |
| Rat-ALP | Forward GGACAATGAGATGCCGCCAGAG  Reverse CCGAGAGGGAAGGGTCAGTCAG |
| Rat-Collagen I | Forward TCACACCTACCTGGACTCATATCTG  Reverse AGGAGGAAGAGGAGGAGGAAGAG |
| Rat-BMP2 | Forward CTTGCCGCCGCCTCCAG  Reverse TGCCTTTCTTCACCTCCTCCTTC |

**Table S2.** Primers of housekeeping gene GAPDH and targeted genes.

**
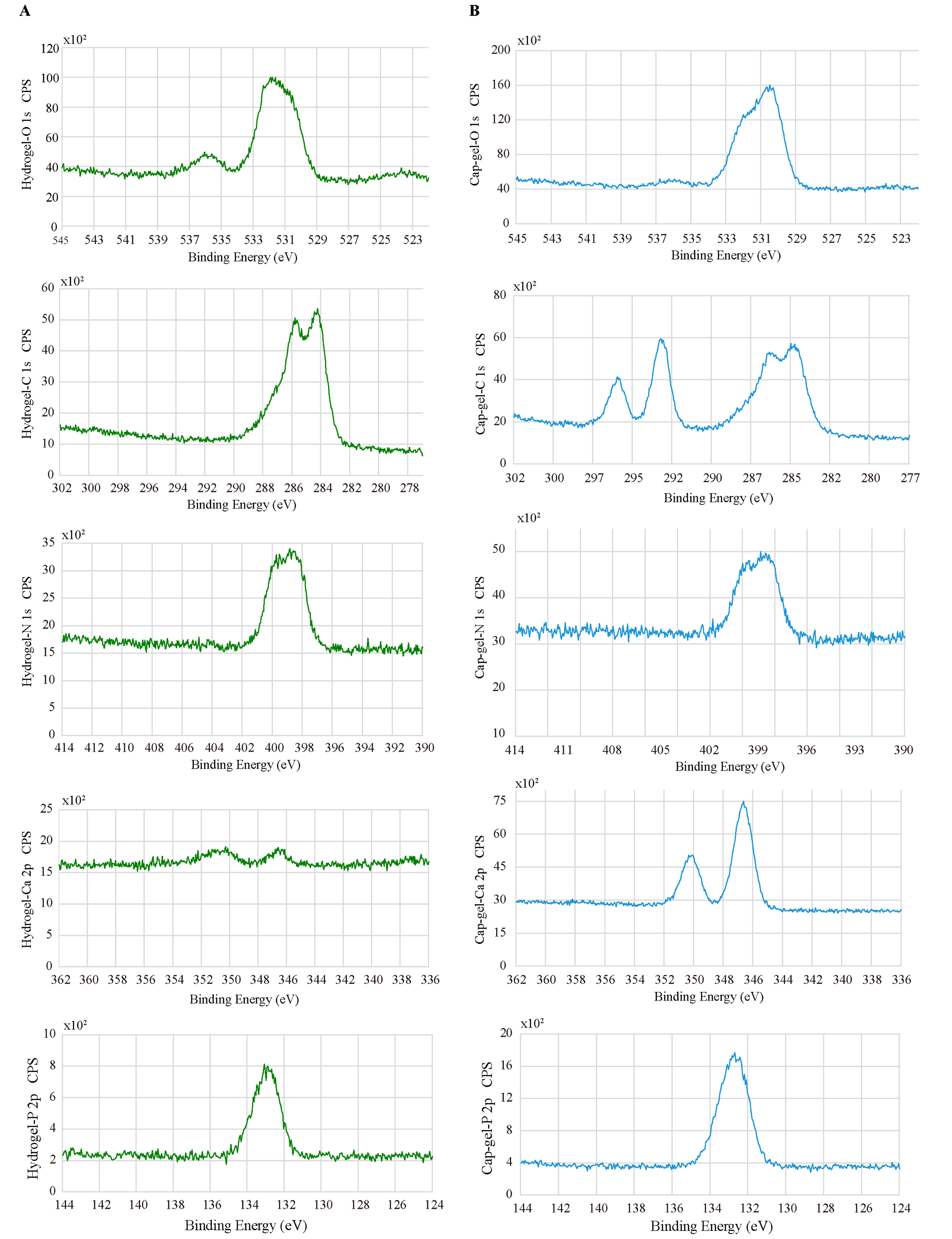
**

**Figure S1. High-resolution XPS spectra of the Hydrogel (green) and Cap-gel (blue) samples.** A) The spectral splitting of Hydrogel. B) The spectral splitting of Cap-gel.

**
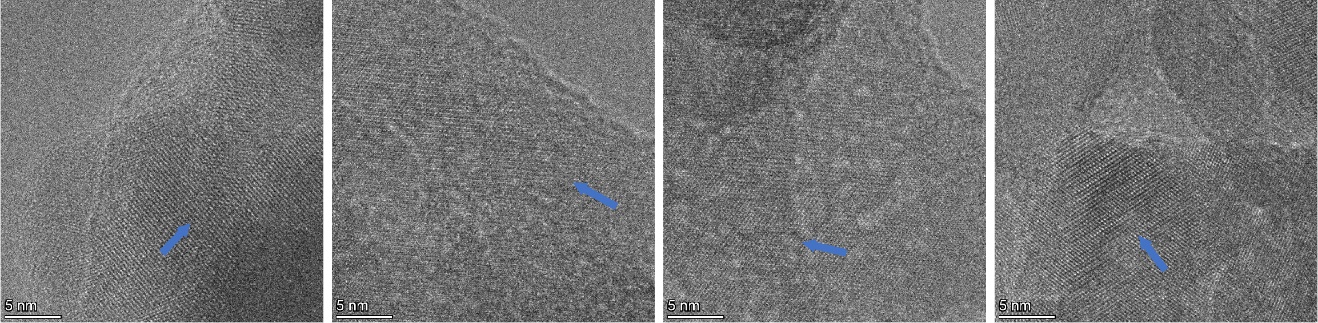
**

**Figure S2.** **HRTEM** **images of Cap-gel showing different crystal lattice spacings.（**Scale bar, 5 nm**）.**

**
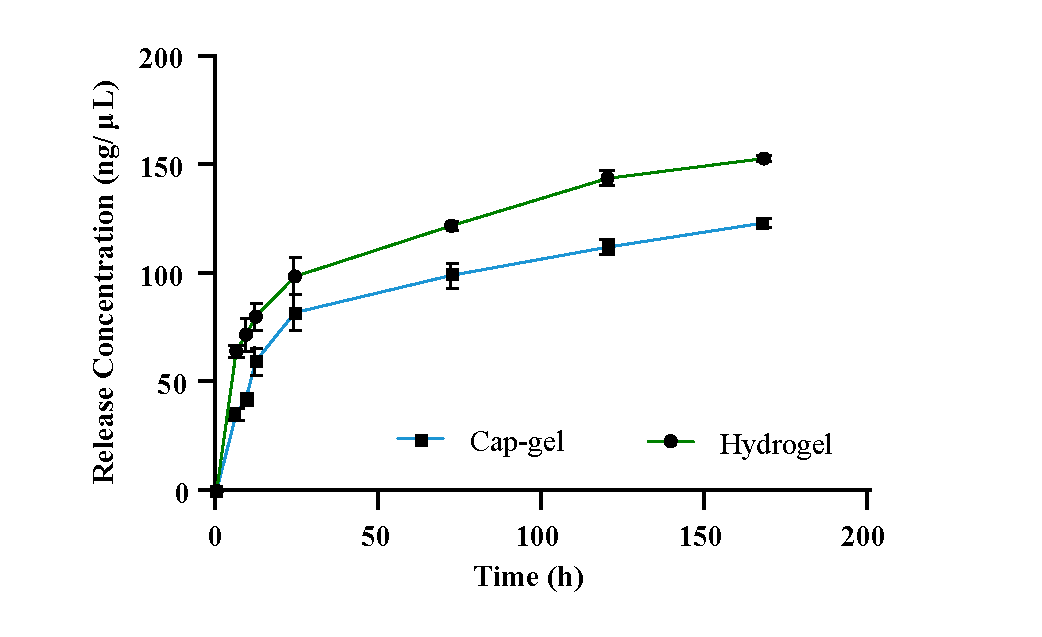
**

**Figure S3.** **Degradation curves of DNA in the Hydrogel and Cap-gel groups in vitro.**

**
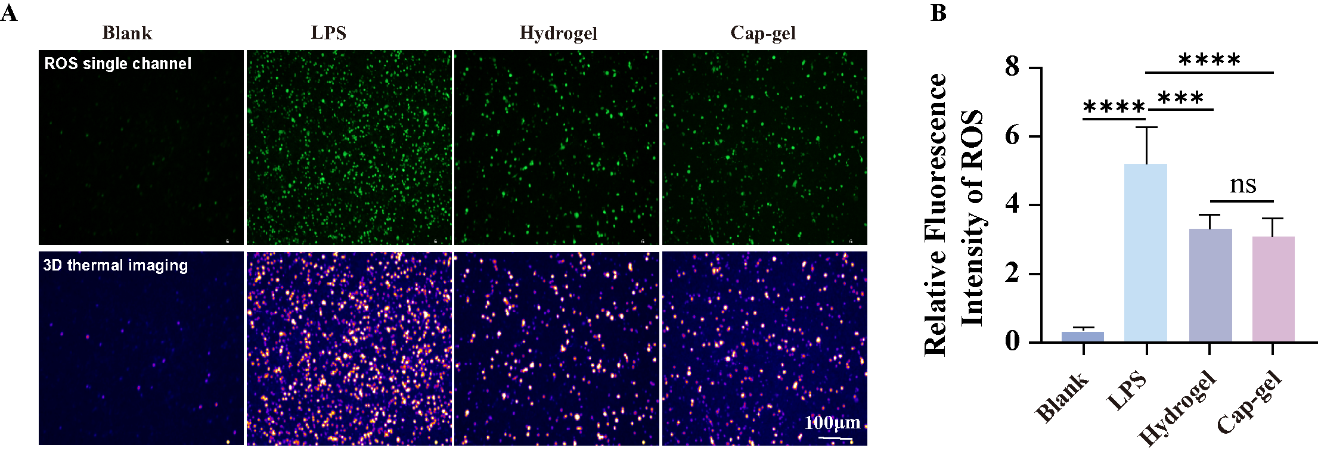
**

**Figure S4. Hydrogel and Cap-gel alleviate the accumulation of intracellular ROS. A)** Fluorescent images of intracellular ROS content. Scale bar, 100 μm. B). Statistical results of the average fluorescence intensity in (A).


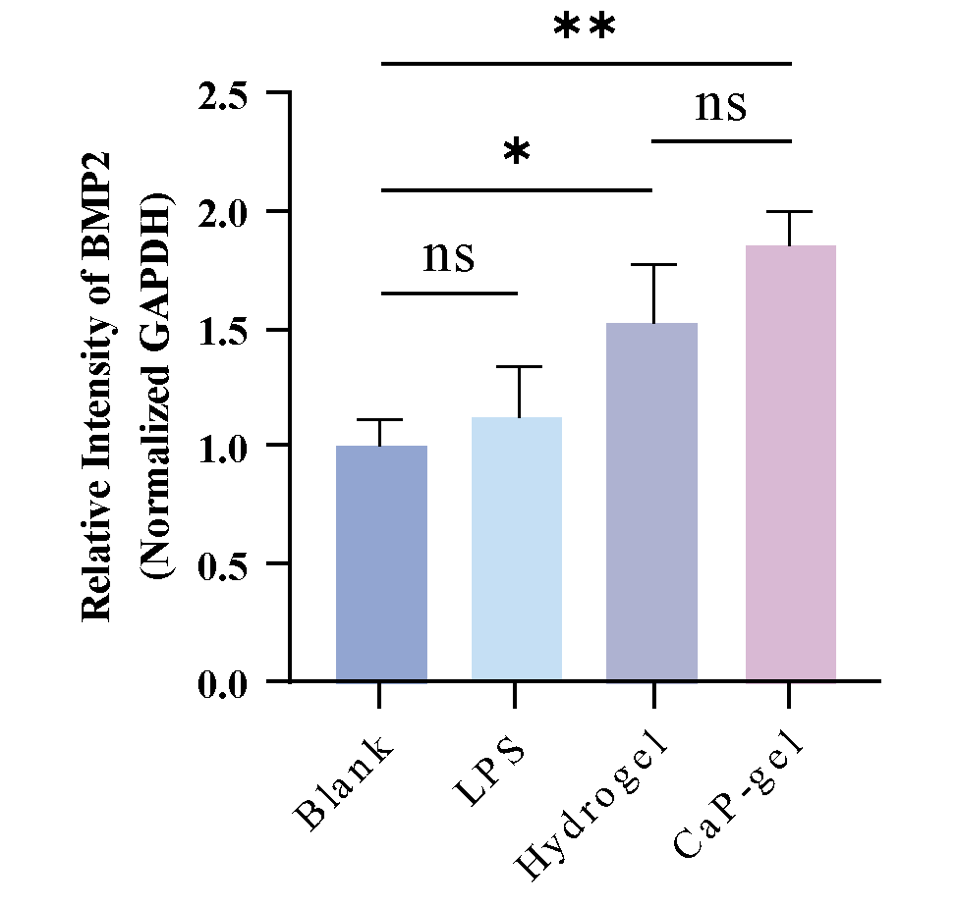


**Figure S5.** **Statistical analysis of the relative expression levels of BMP2 normalized to GAPDH in Figure 2I.** Data are presented as mean ± SD (n ≥ 3), p-values are calculated using one-way ANOVA, *p < 0.05, **p < 0.01, ns: no significance


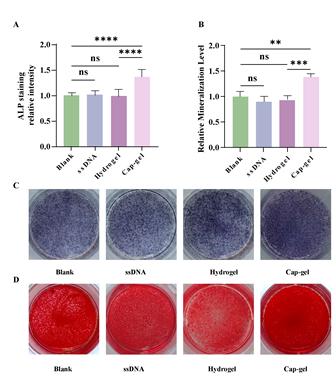


**Figure S6. Statistical analysis of ALP staining and Alizarin Red staining of Figure 3.** A) Statistical analysis chart of Figure 3D. B) Semi-quantitative mineralization levels of calcium nodules in Figure 3E. Data are presented as mean ± SD (n = 3), p-values are calculated using one-way ANOVA, **p < 0.01, ***p < 0.001, ****p < 0.0001, ns: no significance. C) The complete image of the corresponding orifice plate in Figure 3D. D) The complete image of the corresponding orifice plate in Figure 3E.


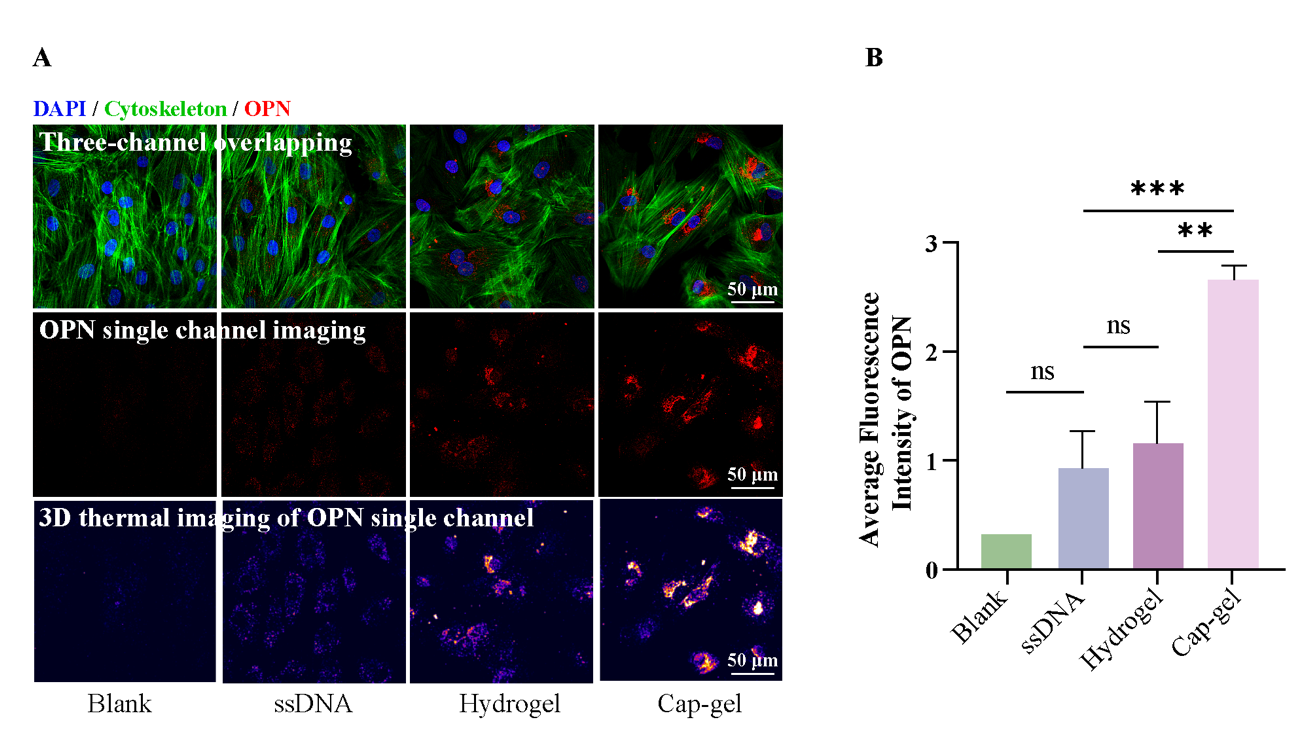


**Figure S7. Cap-gel directly promotes the expression of OPN in vitro.** A) Immunofluorescence detection images of OPN in BMSCs (scale bar: 50 μm). B) Statistical results of relative fluorescence intensity of OPN proteins in (A). Statistical significance was determined by a one-way ANOVA with Sidak. post hoc test. **p < 0.01, ***p < 0.001.

**
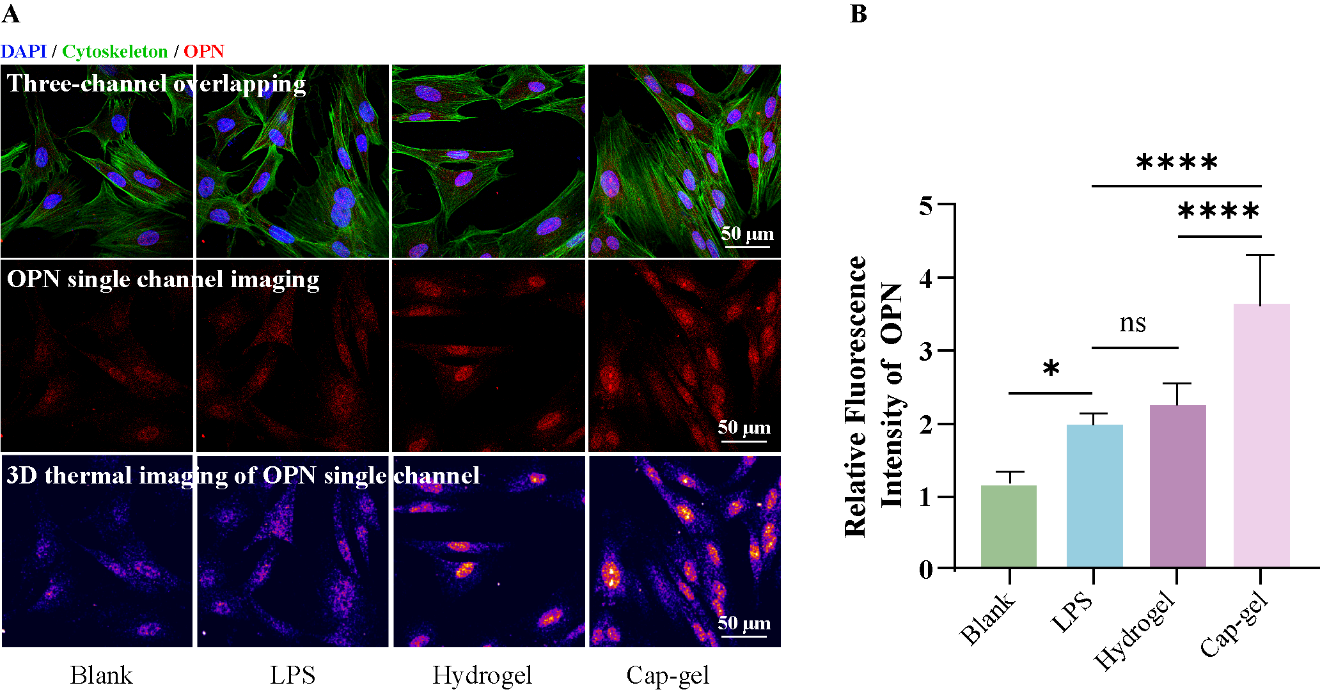
**

**Figure S8. Cap-gel indirectly promotes the expression of OPN in vitro.** A) Immunofluorescence detection images of OPN in BMSCs (scale bar: 50 μm). B) Statistical results of relative fluorescence intensity of OPN proteins in (A). Data are presented as mean ± SD (n = 3), p-values are calculated using one-way ANOVA, *p < 0.05, ****p < 0.0001, ns: no significance.


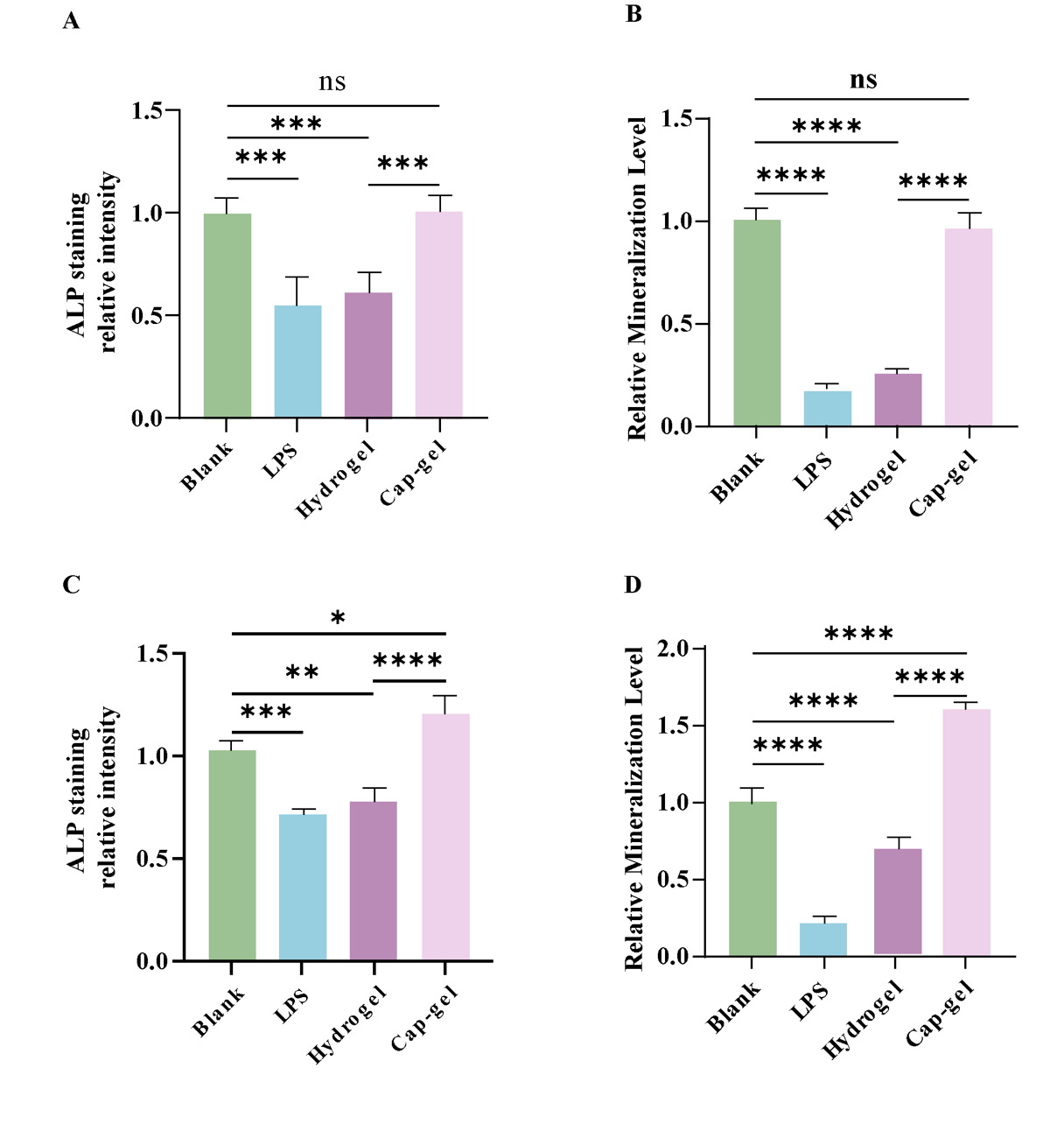


**Figure S9**. **Statistical analysis of ALP staining and Alizarin Red staining of Figure 4.** A) Statistical analysis chart of Figure 4K. B) Semi-quantitative mineralization levels of calcium nodules in Figure 4L C). Statistical analysis chart of Figure 4M. D) Semi-quantitative mineralization levels of calcium nodules in Figure 4N. Data are presented as mean ± SD (n = 3), p-values are calculated using one-way ANOVA, *p < 0.05, **p < 0.01, ***p < 0.001, ****p < 0.0001, ns: no significance.


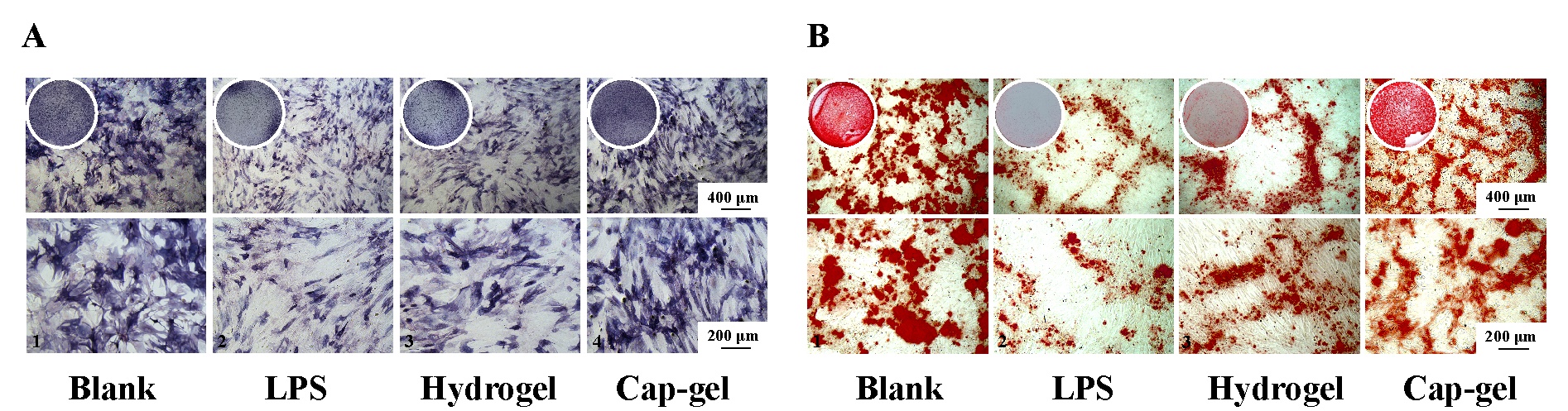


**Figure S10.** **The** **Alkaline phosphatase staining results (A) and Alizarin red staining results (B) after 14 days,** scale bars: 400 μm (upper row), 200 μm (lower row). The complete image of the corresponding orifice plate is placed in the upper left corner.

**
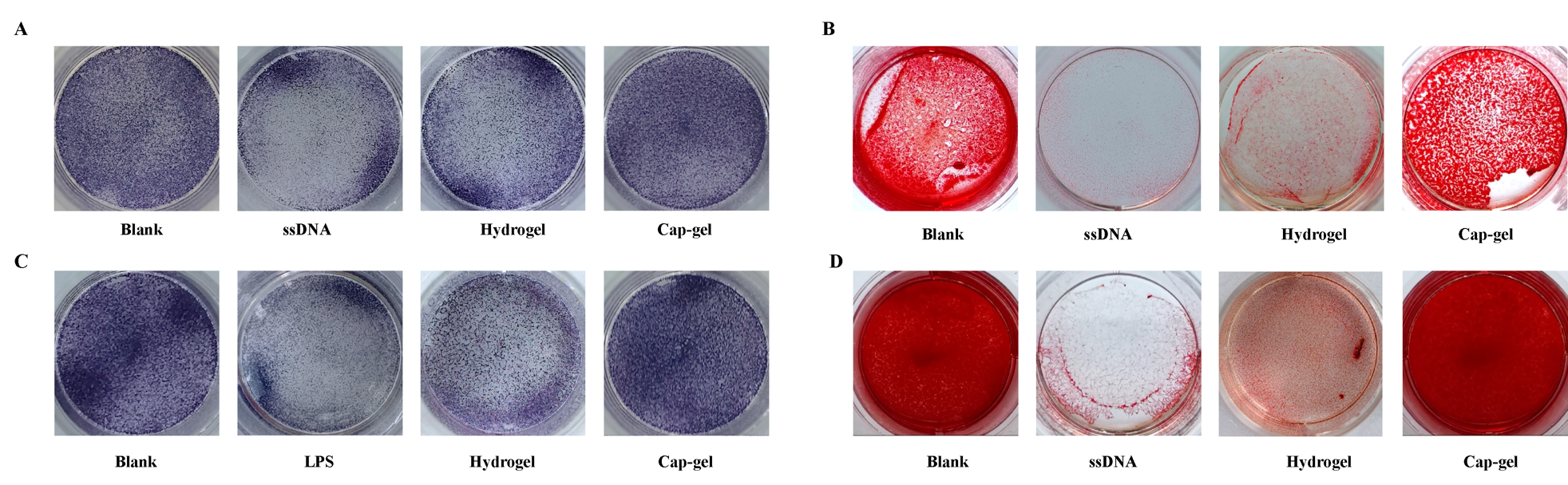
**

**Figure S11. The complete image of ALP staining and Alizarin Red staining of Figure 4.** A) The complete image of the corresponding orifice plate in Figure S10. B) The complete image of the corresponding orifice plate in Figure S10. C) The complete image of the corresponding orifice plate in Figure 4K. D) The complete image of the corresponding orifice plate in Figure 4L.

**
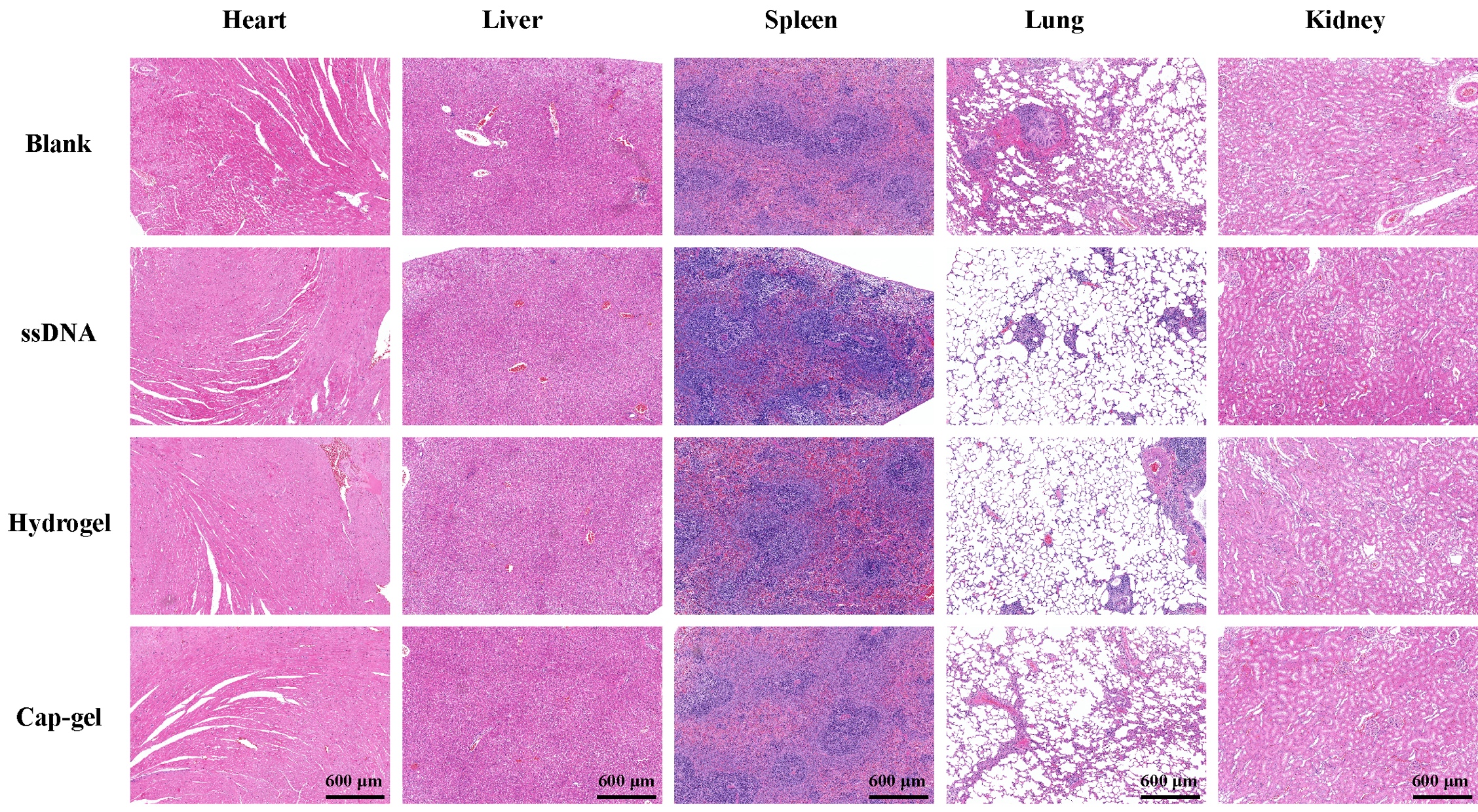
**

**Figure S12. HE examination of rat organs 8 weeks after implantation of different material groups** (scale bar: 600 μm).

**
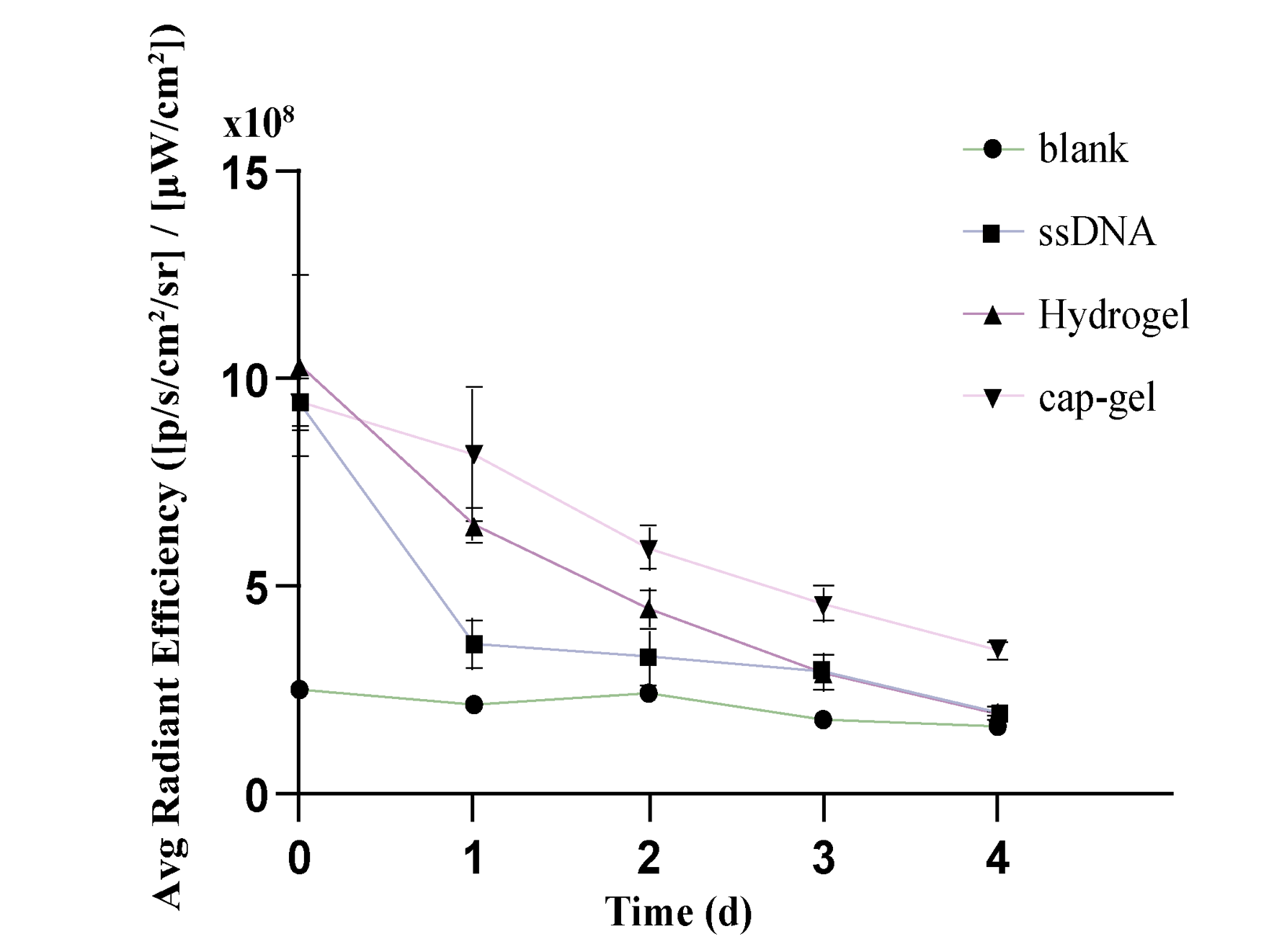
**

**Figure S13. Average fluorescence intensity curves for in vivo imaging in Figure 5C.**

**
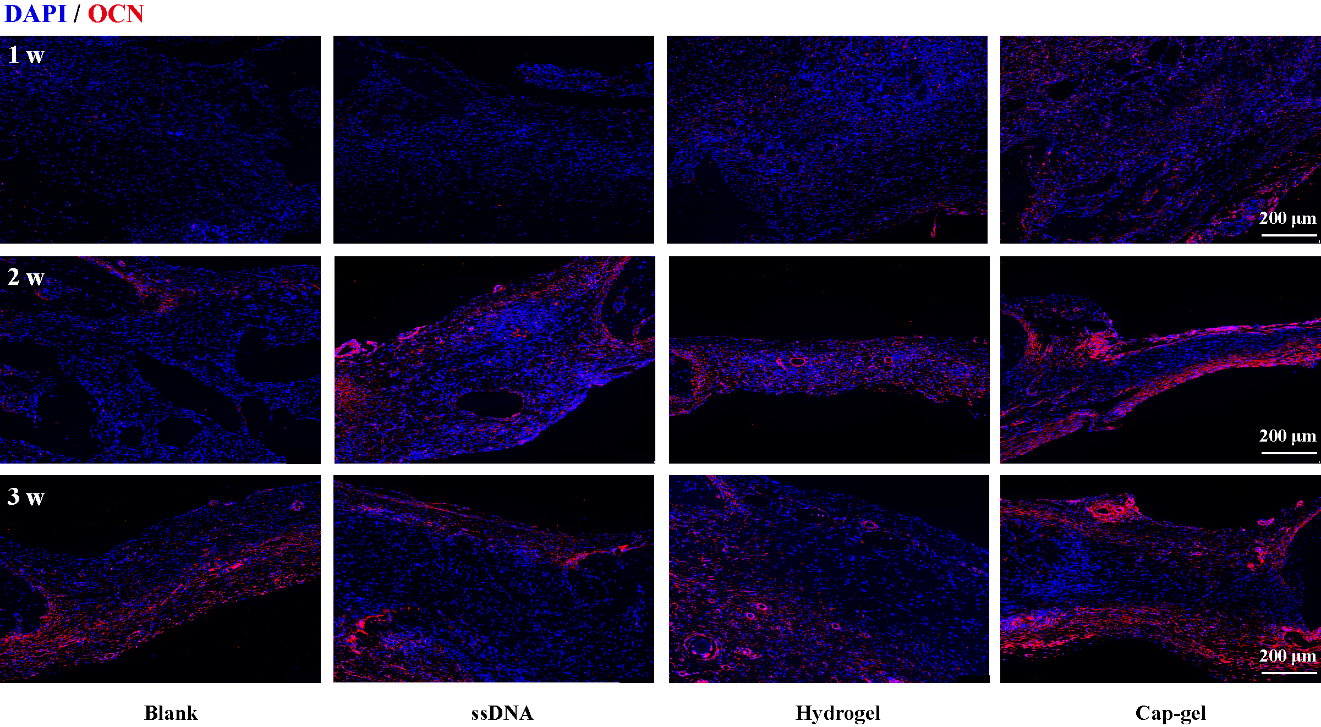
**

**Figure S14.** **Immunofluorescence staining of OCN at 1-3 weeks** (scale bar: 200 μm).

**References**

1.Li, Y., Chen, R., Zhou, B., Dong, Y.&Liu, D. Rational Design of DNA Hydrogels Based on Molecular Dynamics of Polymers. *Adv Mater.* **36**, e2307129 (2024).

2.Liu, Z., Chen, X., Xu, Z., Li, S., Ma, W., et al. Diamond-Inspired DNA Hydrogel Based on Tetrahedral Framework Nucleic Acids for Burn Wound Healing. *Adv Mater.* **37**, e09727 (2025).
